# Risk factors for the rupture of intracranial aneurysms: a systematic review and meta-analysis

**DOI:** 10.3389/fneur.2023.1268438

**Published:** 2023-12-11

**Authors:** Jinyuan Ma, Yuehua Zheng, Puxian Li, Tao Zhou, Zhen Sun, Tongze Ju, Aijun Li

**Affiliations:** ^1^Department of Neurosurgery, Qingdao Binhai University Affiliated Hospital, Qingdao, China; ^2^Department of Neurosurgery, Weifang People’s Hospital Shandong Province, Weifang, China

**Keywords:** risk factors, rupture, intracranial aneurysms, systematic review, meta-analysis

## Abstract

**Purpose:**

The study aimed to identify potential risk factors for aneurysm rupture by performing a systematic review and meta-analysis.

**Materials and methods:**

We systematically searched the PubMed, Embase, and Cochrane Library electronic databases for eligible studies from their inception until June 2023.

**Results:**

Eighteen studies involving 17,069 patients with unruptured intracranial aneurysm (UIA) and 2,699 aneurysm ruptures were selected for the meta-analysis. Hyperlipidemia [odds ratio (OR): 0.47; 95% confidence interval (CI): 0.39–0.56; *p* < 0.001] and a family history of subarachnoid hemorrhage (SAH) (OR: 0.81; 95% CI: 0.71–0.91; *p* = 0.001) were associated with a reduced risk of aneurysm rupture. In contrast, a large-size aneurysm (OR: 4.49; 95% CI: 2.46–8.17; *p* < 0.001), ACA (OR: 3.34; 95% CI: 1.94–5.76; *p* < 0.001), MCA (OR: 2.16; 95% CI: 1.73–2.69; *p* < 0.001), and VABA (OR: 2.20; 95% CI: 1.24–3.91; *p* = 0.007) were associated with an increased risk of aneurysm rupture. Furthermore, the risk of aneurysm rupture was not affected by age, sex, current smoking, hypertension, diabetes mellitus, a history of SAH, and multiple aneurysms.

**Conclusion:**

This study identified the predictors of aneurysm rupture in patients with UIAs, including hyperlipidemia, a family history of SAH, a large-size aneurysm, ACA, MCA, and VABA; patients at high risk for aneurysm rupture should be carefully monitored.

**Systematic Review Registration:**

Our study was registered in the INPLASY platform (INPLASY202360062).

## 1 Introduction

Unruptured intracranial aneurysm (UIA) was considered a “ticking time bomb” and always diagnosed at routine checkups, with an estimated prevalence of 2.3% to 3.2% in the general population (1, 2). The rupture of an intracranial aneurysm can cause aneurysmal subarachnoid hemorrhage (aSAH), with an annual incidence of nearly 9 per 100,000 cases of UIA and 1.4% of UIA rupture annually (3–5). Considering that aSAH is a serious complication of UIA, and the mortality rate has reached 67%, with nearly 50% of the survivors remaining disabled, clinicians should elucidate the risk of rupture (6). Although UIA can be prophylactically treated to prevent aneurysm rupture, nearly 5% of patients are at risk of complications (7). Therefore, in the management of UIAs, the risk of rupture should be balanced with the risk of UIA.

The 5 years risk of aneurysm rupture in UIA can be evaluated using the PHASES scores, which are based on geographic location, hypertension, age, a history of aSAH, and the aneurysm size and location (5). However, the analysis of risk factors based on PHASES scores was restricted because the analysis relies on published articles. Numerous patient and aneurysm characteristics are associated with the rupture risk or are hypothesized to predispose patients to rupture (8). Follow-up imaging could be used to monitor UIA growth, and preventive aneurysm treatment should be administered when aneurysmal growth is observed (7). Several studies have addressed the predictors of aneurysm rupture in patients with UIA; however, these studies only focused on a single factor (5, 9–11). Additional risk factors should be identified to further prevent the aneurysm rupture risk.

In this study, we aimed to identify the risk factors for aneurysm rupture in patients with UIA by conducting a systemic review and meta-analysis.

## 2 Methods

### 2.1 Literature search and selection criteria

This study was conducted and reported following the meta-analysis of observational studies in epidemiology protocol (12), which indicates that the predictors of aneurysm rupture in patients with UIA were eligible for our study, and no restrictions were placed on the publication language and status. Our study was registered in the INPLASY platform (INPLASY202360062). We systematically searched the PubMed, Embase, and Cochrane Library databases for potentially eligible studies through June 2023 using the following search terms: [intracranial aneurysm(s) OR cerebral aneurysm(s)] AND (risk of rupture OR aneurysm rupture OR risk factors OR rupture OR unruptured OR subarachnoid hemorrhage) AND (follow-up OR natural history OR natural course). Reference lists of relevant reviews and original articles were manually searched to identify eligible studies that met the inclusion criteria.

Two reviewers independently performed the literature search and study selection. Any disagreements between the reviewers were resolved by a group discussion until a consensus was reached. The inclusion criteria were as follows:

1. Patients: all of the patients diagnosed with UIA.
2. Exposure: the predictors for aneurysm rupture reported ≥3 times.
3. Outcomes: the study should report the effect estimate for the risk of aneurysm rupture or data could transform into effect estimate.
4. Study design: no restriction for study design, including prospective and retrospective studies.

### 2.2 Data extraction and quality assessment

Two reviewers performed data collection and quality assessment including the first author’s surname, publication year, region, study design, sample size, mean age, male proportion, disease status, a family history of aSAH, hypertension, diabetes mellitus (DM), smoking, location of UIA, follow-up, and the number of aneurysm ruptures. The methodological quality of the included studies was assessed using the Newcastle–Ottawa scale (NOS), which was partially validated to assess the quality of observational studies in the meta-analysis (13). The NOS contained selection (four items), comparability (one item), and outcome (three items), and the “star system” for each study ranged from 0 to 9. Inconsistent results between the reviewers were resolved by an additional reviewer by referring to the original article.

### 2.3 Statistical analysis

The risk factors for aneurysm rupture in each study were assigned odds ratios (ORs) with 95% confidence intervals (CIs), and pooled analysis was performed using the random-effects model considering the underlying variations among the included studies (14, 15). Heterogeneity among the included studies was assessed using *I*^2^ and Cochran Q statistics, and significant heterogeneity was defined as *I*^2^ ≥ 50.0% or a *p*-value of <0.10 (16, 17). The robustness of the pooled conclusions was assessed using sensitivity analysis through the sequential removal of a single study (18). Subgroup analyses were performed according to region, study design, follow-up, and study quality, and the differences between subgroups were compared using the interaction *P* test (19). Funnel plots and Egger–Begg test results were used to assess publication bias (20, 21). The inspection level for pooled effect estimates was two-sided, and a *p*-value of <0.05 was considered statistically significant. The STATA software (version 12.0; Stata Corporation, College Station, TX, United States) was used for all statistical analyses.

## 3 Results

### 3.1 Literature search

**Figure 1:**
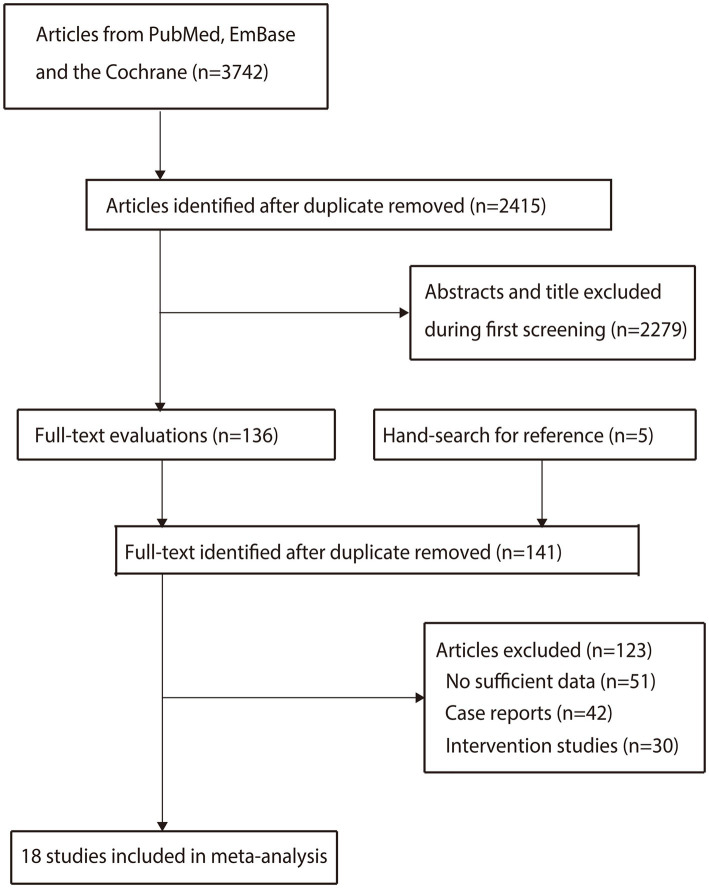
Literature search and study selection.

We initially identified 3,742 articles from the electronic searches, and 2,415 articles were retained after duplicates were removed. Subsequently, 2,279 studies were excluded owing to irrelevant titles or abstracts. Five additional articles were identified by reviewing the reference lists of the relevant reviews and original articles. A total of 141 studies were retrieved for detailed evaluation, and 123 studies were excluded because of insufficient data (*n* = 51), case reports (*n* = 42), or intervention studies (*n* = 30). The remaining 18 studies were included in the final meta-analysis (Figure 1) (22–39).

### 3.2 Characteristics of the included studies

The baseline characteristics of the identified studies and patients with UIA are summarized in Table 1. In total, 17,069 patients with UIA were included, and the sample sizes ranged from 70 to 5,720. Eight studies were designed as prospective cohorts, and the remaining eight were designed as retrospective cohorts. Nine studies were conducted in Western countries, eight were performed in Eastern countries, and the remaining one was conducted in multiple countries. These studies reported 2,699 aneurysm ruptures. The methodological quality of the individual studies was assessed using the NOS as follows: six studies with eight stars, seven studies with seven stars, and five studies with six stars. Studies with 7–9 stars were regarded as high quality, while 4–6 stars were considered moderate quality.

**Table 1:** Baseline characteristics of included studies and involved patients.

Study	Region	Study design	Sample size	Age (years)	Male (%)	No. of UIA	Family history of aSAH	Hypertension (%)	DM (%)	Smoking (%)	Location of UIA	Follow-up	No. of aneurysm rupture	NOS scale
Ishibashi et al. (22)	Japan	Pro	419	60.8	33.0	529	NA	NA	NA	NA	ICA (41%), ACA (20%), MCA (27%), VABA (12%)	2.5 years	19	8
Sonobe et al. (23)	Japan	Pro	374	61.9	36.4	448	8.3	24.9	6.1	NA	ICA (38.6%), MCA (35.3%), Acom (13.4%), distal ACA (2.7%), BA (7.4%), VA (0.9%)	3.5 years	18	8
Morita et al. (24)	Japan	Pro	5,720	62.5	33.5	6,697	12.9	43.4	6.3	NA	MCA (36.2%), Acom (15.5%), ICA (18.6%), ICPcom (15.5%), BA (6.6%), VA (1.8%)	1.7 years	111	8
Guresir et al. (25)	Germany	Pro	263	55.0	22.4	384	3.0	42.6	5.3	52.1	ACA (46%), MCA (33%), Acom (14%), distal ACA (7%)	4.0 years	3	7
Juvela et al. (26)	Finland	Pro	142	41.8	46.0	181	NA	36.0	NA	47.0	ICA (42%), ACA (4%), Acom (6%), MCA (45%), VABA (3%)	21.0 years	34	7
Korja et al. (27)	Finland	Pro	118	43.5	48.3	146	NA	25.0	NA	39.0	NA	13.6 years	38	7
Gross et al. (28)	United States	Retro	747	53.9	17.0	1,013	NA	39.0	NA	32.0	Acom (17%), MCA (16%), VA (5%), BA (8%), ICA (6%)	7.0 years	303	6
Hishikawa et al. (29)	Japan	Pro	1,896	74.3	27.1	2,227	9.3	53.6	NA	8.1	MCA (33.9%), Acom (17.9%), ICA (11.6%), ICPcom (19.9%), BA (8.7%), VA (2.6%)	2.2 years	68	7
Murayama et al. (30)	Japan	Pro	2,252	65.0	32.4	2,897	NA	46.5	5.7	13.5	MCA (27.3%), ACA (16.8%), ICA (26.8%), ICPcom (20.5%), VABA (8.6%)	2.6 years	56	8
Teo et al. (31)	United Kingdom	Retro	94	53.0	21.3	152	NA	NA	NA	NA	MCA (38%), ICA (21%), Pcom (16%), Acom (9%), BA (7%)	3.4 years	4	6
Mocco et al. (32)	United States	Pro	255	NA	23.9	NA	NA	NA	NA	NA	ICA (25.5%), ACA/Acom (5.9%), MCA (14.9%), BA (18.0%), Pcom (26.3%), PCA (2.4%)	7.0 years	57	7
Hostettler et al. (33)	United Kingdom	Retro	2,334	54.2	29.7	2,942	12.4	35.3	4.6	42.5	MCA (22.9%), ICA (12.8%), ACA/Acom (24.5%), Pcom (18.1%), PCA (11.4%)	4.0 years	1,729	8
Funakoshi et al. (34)	Japan	Retro	595	63.9	27.1	595	NA	NA	NA	NA	ICA (58.2%), MCA (1.7%), Acom (20.7%), VA (3.9%), BA (11.9%)	6.2 years	169	6
Wang et al. (35)	China	Pro	1,087	60.3	53.3	1,087	NA	53.4	19.9	21.4	ICA (65.6%), MCA (8.5%), ACA (3.1%), Acom (7.2%), Pcom (4.0%), BA (4.3%), VA (3.3%), PCA (2.1%)	2.8 years	11	8
van der Kamp et al. (36)	Canada, Europe, China, and Japan	Retro	312	61.0	29.0	329	NA	NA	NA	41.0	ICA (25%), MCA (32%), Pcom (8%), Acom (15%), ACA (5%), BA (8%), VABA (7%)	2.8 years	24	7
Lee et al. (37)	Korea	Retro	117	52.8	57.3	117	4.3	42.7	11.1	28.2	VA (100%)	3.0 years	34	6
Dmytriw et al. (38)	Canada	Retro	70	51.7	55.7	78	NA	54.2	10.9	30.9	VA (38.5%), BA (30.8%), PCA (19.2%), PICA (10.3%), AICA (1.3%)	3.0 years	6	6
Spencer et al. (39)	UK	Retro	274	54.8	24.1	445	17.2	45.3	7.3	55.1	MCA (74.5%), ICA (24.8%), Acom (17.5%), Pcom (13.1%), ACA (5.8%), BA (5.8%), PCA (2.6%)	3.8 years	15	7

ACA, anterior cerebral artery; Acom, anterior communicating artery; BA, basilar artery; DM, diabetes mellitus; ICA, internal carotid artery; ICPcom, internal carotid-posterior communicating artery; MCA, middle cerebral artery; NA, not available; PCA, posterior cerebral artery; Pro, prospective; Retro, retrospective; UIA, unruptured intracranial aneurysm; VA, vertebral artery; VABA, vertebrobasilar artery.

### 3.3 Age and sex

**Figure 2:**
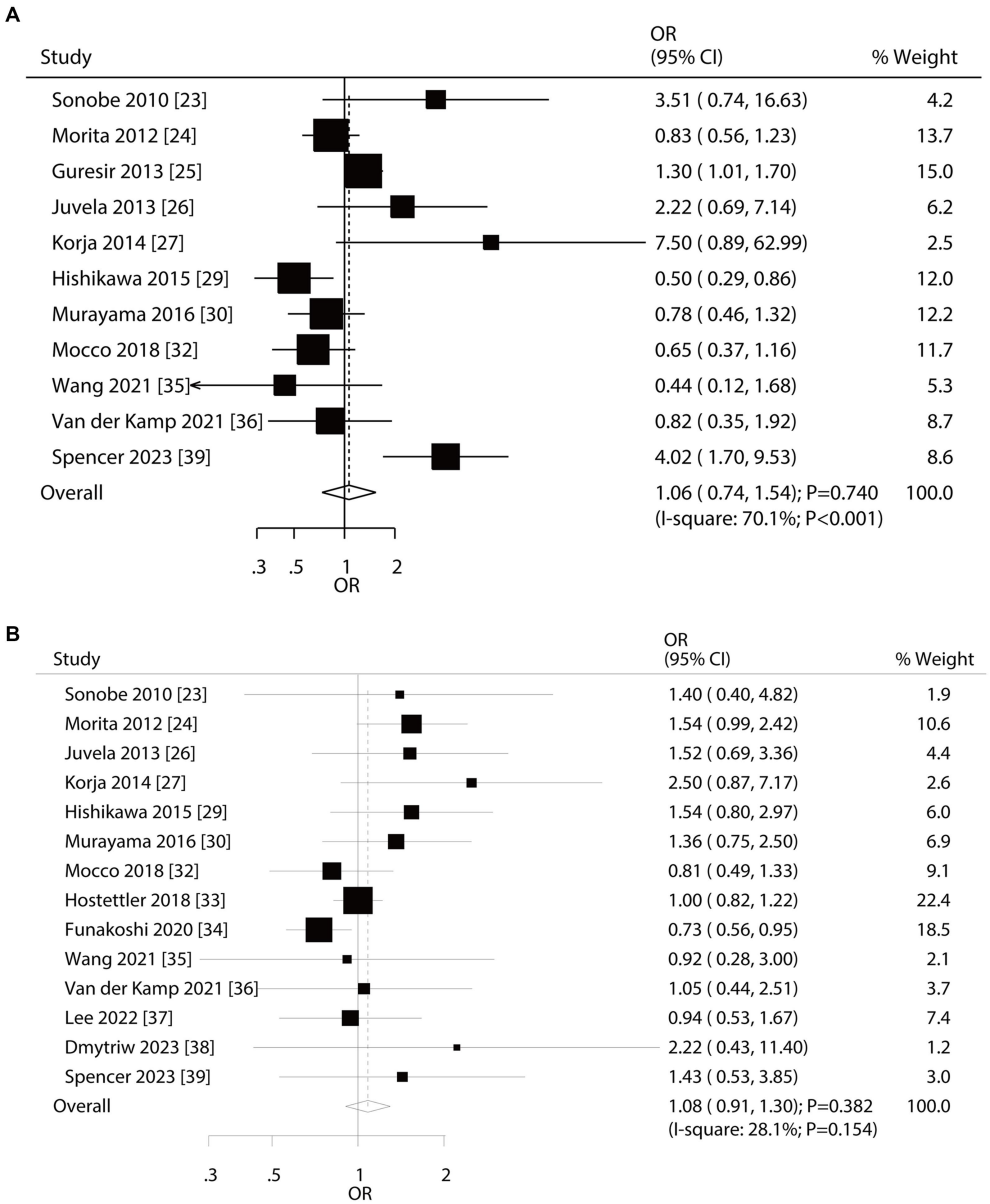
Effect of age and sex on the risk of aneurysm rupture in patients with unruptured intracranial aneurysms (UIA). **(A)** Younger vs. elder. **(B)** Female vs. male.

A total of 11 and 14 studies reported the roles of age and sex on the risk of aneurysm rupture in UIA patients, respectively (Figure 2). Age (OR: 1.06; 95% CI: 0.74–1.54; *p* = 0.740) and sex (OR: 1.08; 95% CI: 0.91–1.30; *p* = 0.382) were not associated with the aneurysm rupture risk in patients with UIA. There was significant heterogeneity in age (*I*^2^ = 70.1%; *p* < 0.001); in contrast, no significant heterogeneity was observed in sex (*I*^2^ = 28.1%; *p* = 0.154). Sensitivity analyses indicated that the pooled conclusions regarding the roles of age and sex were not changed by the sequential removal of individual studies (Supplementary File 1). Subgroup analyses indicated that younger patients had a reduced risk of aneurysm rupture compared to older patients if the follow-up duration was <3.0 years and the role of age in the risk of aneurysm rupture could be affected by region (*p* = 0.002) and follow-up (*p* < 0.001) (Table 2). No evidence of publication bias for age was observed (*p*-value for Egger: 0.622; *p*-value for Begg: 0.350; Supplementary File 2). Although the Begg test indicated no significant publication bias for sex (*p* = 0.324), a potentially significant publication bias was observed using the Egger test (*p* = 0.035) (Supplementary File 2).

**Table 2:** Subgroup analyses for the risk factors of aneurysm rupture in UIA patients.

Outcomes	Factors	Subgroups	No. of studies	OR and 95% CI	*p* value	*I*^2^ (%)	*Q* statistic	Interaction *p*-value
Age (younger vs. elder)	Region	Eastern	5	0.74 (0.50–1.09)	0.128	41.2	0.147	0.002
		Western	6	1.47 (0.84–2.56)	0.173	70.1	0.005	
	Study design	Prospective	9	0.94 (0.65–1.35)	0.727	65.5	0.003	0.057
		Retrospective	2	1.81 (0.38–8.61)	0.454	84.9	0.010	
	Follow-up (years)	≥3.0	6	1.82 (0.99–3.37)	0.056	70.8	0.004	<0.001
		<3.0	5	0.71 (0.55–0.92)	0.010	0.0	0.568	
	Study quality	High	11	1.06 (0.74–1.54)	0.740	70.1	<0.001	—
		Moderate	0	—	—	—	—	
Gender (female vs. male)	Region	Eastern	7	1.11 (0.81–1.52)	0.501	50.0	0.062	0.653
		Western	7	1.04 (0.88–1.23)	0.647	0.0	0.438	
	Study design	Prospective	8	1.31 (1.03–1.66)	0.026	0.0	0.511	0.012
		Retrospective	6	0.92 (0.77–1.09)	0.321	10.4	0.349	
	Follow-up (years)	≥3.0	9	0.98 (0.79–1.20)	0.821	27.9	0.197	0.016
		<3.0	5	1.39 (1.05–1.86)	0.024	0.0	0.883	
	Study quality	High	11	1.12 (0.97–1.30)	0.128	0.0	0.494	0.011
		Moderate	3	0.80 (0.60–1.06)	0.119	9.6	0.331	
Current smoker	Region	Eastern	5	1.01 (0.52–1.95)	0.988	59.1	0.044	0.273
		Western	7	1.40 (1.13–1.72)	0.002	2.9	0.404	
	Study design	Prospective	6	1.33 (0.63–2.83)	0.456	67.5	0.009	0.948
		Retrospective	6	1.31 (1.11–1.56)	0.002	0.0	0.883	
	Follow-up (years)	≥3.0	8	1.49 (1.04–2.15)	0.031	36.3	0.139	0.226
		<3.0	4	1.06 (0.58–1.91)	0.858	36.1	0.195	
	Study quality	High	9	1.30 (0.87–1.94)	0.193	52.3	0.033	0.679
		Moderate	3	1.46 (0.87–2.47)	0.153	0.0	0.898	
Hypertension	Region	Eastern	6	1.44 (0.86–2.41)	0.167	74.3	0.002	0.393
		Western	6	1.53 (0.64–3.67)	0.343	95.8	<0.001	
	Study design	Prospective	8	1.75 (1.12–2.73)	0.014	79.3	<0.001	<0.001
		Retrospective	4	1.04 (0.49–2.19)	0.928	81.3	0.001	
	Follow-up (years)	≥3.0	8	1.62 (0.78–3.40)	0.199	94.6	<0.001	0.370
		<3.0	4	1.31 (0.79–2.16)	0.292	67.1	0.028	
	Study quality	High	10	1.66 (0.94–2.93)	0.079	93.5	<0.001	0.338
		Moderate	2	1.00 (0.36–2.77)	0.994	40.9	0.193	
DM	Region	Eastern	3	0.77 (0.27–2.20)	0.619	35.7	0.211	0.362
		Western	2	1.69 (0.18–15.99)	0.646	88.5	0.003	
	Study design	Prospective	2	0.33 (0.08–1.37)	0.128	0.0	0.760	0.230
		Retrospective	3	1.39 (0.48–4.05)	0.544	81.9	0.004	
	Follow-up (years)	≥3.0	3	1.39 (0.48–4.05)	0.544	81.9	0.004	0.230
		<3.0	2	0.33 (0.08–1.37)	0.128	0.0	0.760	
	Study quality	High	3	0.57 (0.39–0.85)	0.005	0.0	0.703	0.003
		Moderate	2	2.49 (0.61–10.24)	0.205	66.8	0.082	
Hyperlipidemia	Region	Eastern	4	0.59 (0.37–0.93)	0.023	0.0	0.904	0.291
		Western	2	0.45 (0.37–0.55)	<0.001	0.0	0.712	
	Study design	Prospective	3	0.55 (0.33–0.92)	0.022	0.0	0.880	0.499
		Retrospective	3	0.46 (0.38–0.56)	<0.001	0.0	0.575	
	Follow-up (years)	≥3.0	3	0.46 (0.38–0.56)	<0.001	0.0	0.575	0.499
		<3.0	3	0.55 (0.33–0.92)	0.022	0.0	0.880	
	Study quality	High	4	0.46 (0.38–0.56)	<0.001	0.0	0.844	0.322
		Moderate	2	0.75 (0.29–1.91)	0.545	0.0	0.907	
History of SAH	Region	Eastern	4	3.17 (1.51–6.66)	0.002	41.6	0.162	0.005
		Western	3	0.90 (0.42–1.92)	0.778	0.0	0.613	
	Study design	Prospective	4	3.17 (1.51–6.66)	0.002	41.6	0.162	0.005
		Retrospective	3	0.90 (0.42–1.92)	0.778	0.0	0.613	
	Follow-up (years)	≥3.0	3	0.85 (0.34–2.10)	0.728	0.0	0.595	0.021
		<3.0	4	2.75 (1.22–6.23)	0.015	59.8	0.058	
	Study quality	High	6	2.08 (1.03–4.22)	0.041	57.3	0.039	0.143
		Moderate	1	0.28 (0.02–4.45)	0.367	—	—	
Family history of SAH	Region	Eastern	3	0.91 (0.67–1.24)	0.545	0.0	0.654	0.418
		Western	1	0.79 (0.69–0.90)	0.001	—	—	
	Study design	Prospective	3	0.91 (0.67–1.24)	0.545	0.0	0.654	0.418
		Retrospective	1	0.79 (0.69–0.90)	0.001	—	—	
	Follow-up (years)	≥3.0	2	0.83 (0.70–0.98)	0.028	21.4	0.259	0.629
		<3.0	2	0.69 (0.35–1.35)	0.273	0.0	0.983	
	Study quality	High	4	0.81 (0.71–0.91)	0.001	0.0	0.681	—
		Moderate	0	—	—	—	—	
Large size of aneurysm	Region	Eastern	6	7.99 (5.00–12.76)	<0.001	74.2	0.002	<0.001
		Western	6	2.32 (1.19–4.52)	0.013	77.3	0.001	
	Study design	Prospective	9	4.99 (2.41–10.34)	<0.001	93.5	<0.001	0.498
		Retrospective	3	3.53 (2.13–5.86)	<0.001	0.0	0.695	
	Follow-up (years)	≥3.0	6	2.51 (1.21–5.19)	0.013	78.6	<0.001	<0.001
		<3.0	6	7.27 (4.45–11.85)	<0.001	78.5	<0.001	
	Study quality	High	11	4.69 (2.53–8.68)	<0.001	92.0	<0.001	0.407
		Moderate	1	1.62 (0.17–15.18)	0.673	—	—	
Multiple aneurysm	Region	Eastern	4	1.51 (0.99–2.30)	0.056	37.7	0.186	0.041
		Western	3	0.97 (0.79–1.19)	0.755	0.0	0.414	
	Study design	Prospective	5	1.39 (0.93–2.06)	0.107	35.8	0.183	0.084
		Retrospective	2	1.16 (0.54–2.46)	0.703	35.9	0.212	
	Follow-up (years)	≥3.0	4	1.33 (0.73–2.41)	0.354	60.4	0.055	0.303
		<3.0	3	1.28 (0.89–1.85)	0.186	5.9	0.345	
	Study quality	High	6	1.23 (0.89–1.69)	0.214	47.6	0.089	0.267
		Moderate	1	2.55 (0.56–11.64)	0.227	—	—	
ACA vs. ICA	Region	Eastern	6	2.46 (1.58–3.84)	<0.001	22.2	0.267	<0.001
		Western	3	5.35 (3.04–9.43)	<0.001	41.3	0.182	
	Study design	Prospective	6	3.30 (1.91–5.70)	<0.001	13.7	0.327	0.803
		Retrospective	3	3.58 (1.34–9.57)	0.011	92.2	<0.001	
	Follow-up (years)	≥3.0	5	3.95 (1.87–8.33)	<0.001	85.0	<0.001	0.676
		<3.0	4	2.61 (1.18–5.79)	0.018	36.3	0.194	
	Study quality	High	7	4.06 (2.33–7.07)	<0.001	48.7	0.069	<0.001
		Moderate	2	2.14 (1.64–2.80)	<0.001	0.0	0.662	
MCA vs. ICA	Region	Eastern	6	1.96 (1.28–3.02)	0.002	0.0	0.547	0.615
		Western	4	2.23 (1.73–2.89)	<0.001	0.0	0.826	
	Study design	Prospective	6	2.07 (1.32–3.24)	0.001	0.0	0.551	0.831
		Retrospective	4	2.19 (1.70–2.82)	<0.001	0.0	0.770	
	Follow-up (years)	≥3.0	5	2.17 (1.70–2.78)	<0.001	0.0	0.929	0.910
		<3.0	5	2.10 (1.24–3.53)	0.005	6.7	0.368	
	Study quality	High	8	2.14 (1.68–2.72)	<0.001	0.0	0.728	0.849
		Moderate	2	2.27 (1.27–4.08)	0.006	0.0	0.405	
VABA vs. ICA	Region	Eastern	6	2.20 (1.10–4.40)	0.025	53.5	0.044	0.332
		Western	1	2.43 (1.02–5.78)	0.045	—	—	
	Study design	Prospective	6	2.91 (1.78–4.75)	<0.001	0.0	0.586	0.002
		Retrospective	1	1.07 (0.70–1.63)	0.752	—	—	
	Follow-up (years)	≥3.0	3	1.40 (0.78–2.51)	0.262	28.7	0.246	0.009
		<3.0	4	3.22 (1.72–6.03)	<0.001	5.4	0.376	
	Study quality	High	6	2.91 (1.78–4.75)	<0.001	0.0	0.586	0.002
		Moderate	1	1.07 (0.70–1.63)	0.752	—	—	

### 3.4 Smoking status and hypertension

**Figure 3:**
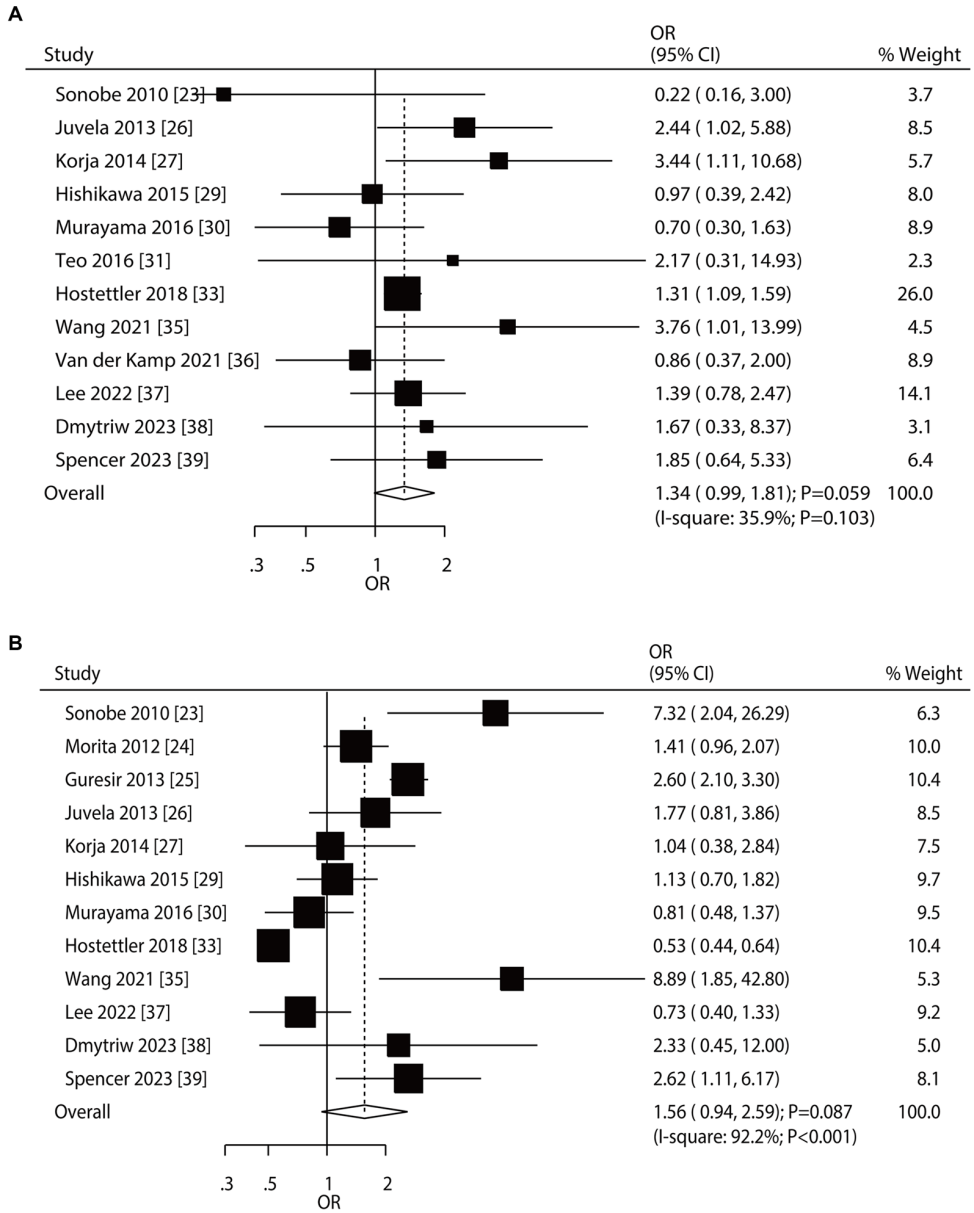
Effect of smoking status and hypertension on the risk of aneurysm rupture in patients with unruptured intracranial aneurysms (UIA). **(A)** Current smoker. **(B)** Hypertension.

Overall, 12 and 12 studies reported the roles of smoking status and hypertension on the risk of aneurysm rupture in UIA patients, respectively (Figure 3). Current smoking (OR: 1.34; 95% CI: 0.99–1.81; *p* = 0.059) and hypertension (OR: 1.56; 95% CI: 0.94–2.59; *p* = 0.087) were not associated with the risk of aneurysm rupture. There was no significant heterogeneity for current smoking (*I*^2^ = 35.9%; *p* = 0.103); in contrast, significant heterogeneity was observed for hypertension (*I*^2^ = 92.2%; *p* < 0.001). Sensitivity analyses revealed that current smoking and hypertension were associated with an elevated risk of aneurysm rupture (Supplementary File 1). Subgroup analyses showed current smoking was associated with an increased risk of aneurysm rupture when pooled studies were conducted in Western countries, studies with a retrospective cohort, and a follow-up of ≥3.0 years. Hypertension induced excess risk of aneurysm rupture in pooled prospective cohort studies. Moreover, the role of hypertension in the risk of aneurysm rupture was affected by the study design (*p* < 0.001) (Table 2). There was no significant publication bias for current smoking (*p*-value for Egger: 0.840; *p*-value for Begg: 0.451) or hypertension (*p*-value for Egger: 0.276; *p*-value for Begg: 0.451; Supplementary File 2).

### 3.5 Diabetes mellitus and hyperlipidemia

**Figure 4:**
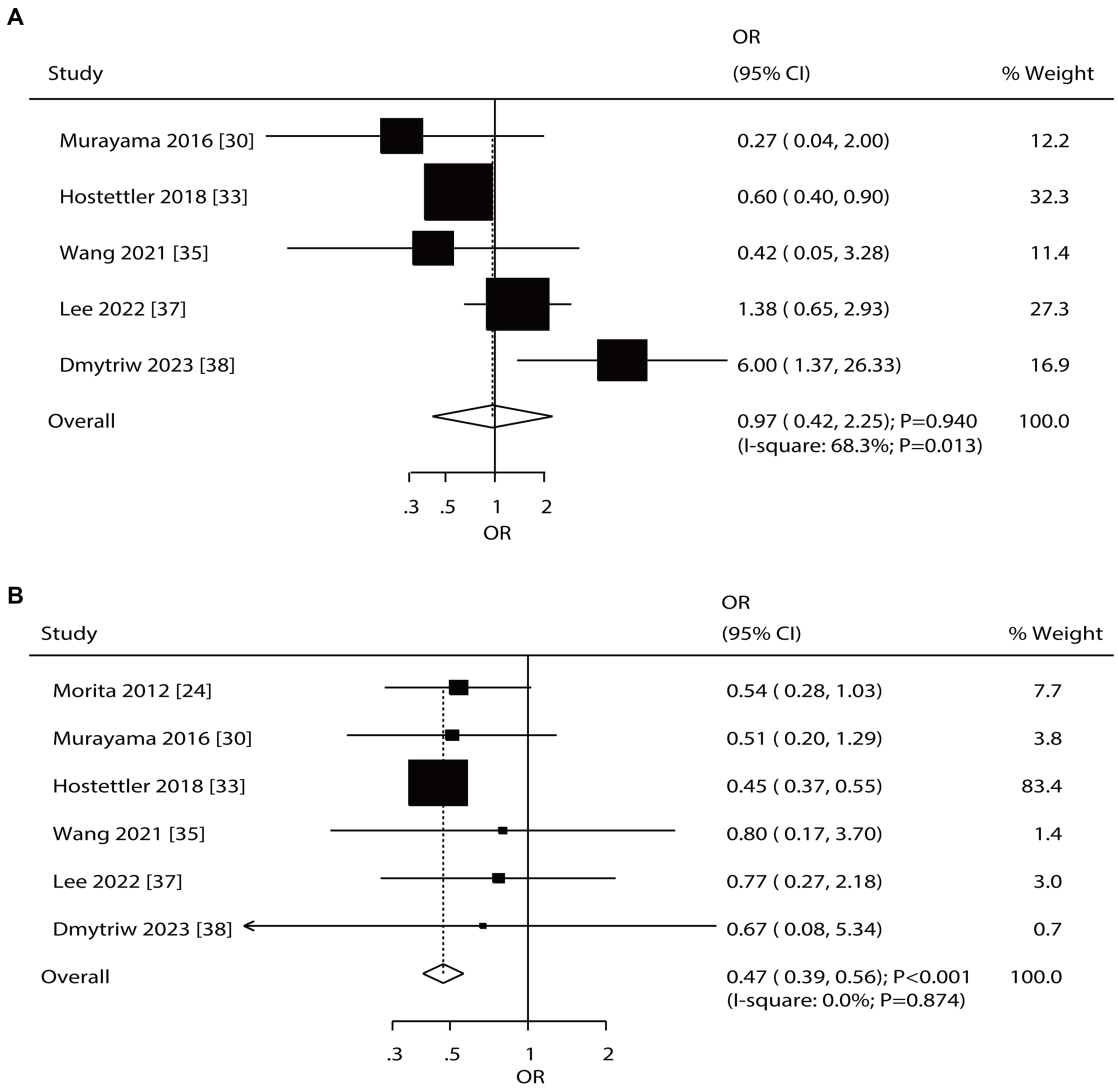
Effect of diabetes mellitus (DM) and hyperlipidemia on the risk of aneurysm rupture in patients with unruptured intracranial aneurysms (UIA). **(A)** DM. **(B)** Hyperlipidemia.

Overall, 5 and 6 studies reported the roles of diabetes mellitus (DM) and hyperlipidemia in the risk of aneurysm rupture in patients with UIA, respectively (Figure 4). Notably, DM was not associated with the risk of aneurysm rupture (OR: 0.97; 95% CI: 0.42–2.25; *p* = 0.940); in contrast, hyperlipidemia was associated with a reduced risk of aneurysm rupture (OR: 0.47; 95% CI: 0.39–0.56; *p* < 0.001). There was significant heterogeneity for DM (*I*^2^ = 68.3%; *p* = 0.013); in contrast, no evidence of heterogeneity was observed for hyperlipidemia (*I*^2^ = 0.0%; *p* = 0.874). Sensitivity analyses indicated that the pooled conclusions for DM and hyperlipidemia were robust after excluding one study (Supplementary File 1). Subgroup analysis indicated that DM was associated with a reduced risk of aneurysm rupture when pooled studies were of high quality, and study quality could affect the role of DM in the risk of aneurysm rupture (*p* = 0.003). Moreover, hyperlipidemia was associated with a lower risk of aneurysm rupture in most subgroups; in contrast, there was no significant association between hyperlipidemia and the risk of aneurysm rupture if the pooled studies were of moderate quality (Table 2). There was no significant publication bias for DM (*p*-value for Egger: 0.619; *p*-value for Begg: 0.806). Although the Begg test indicated no significant publication bias for hyperlipidemia (*p* = 0.452), the Egger test suggested a potentially significant publication bias for hyperlipidemia (*p* = 0.016) (Supplementary File 2).

### 3.6 History of SAH and family history of SAH

**Figure 5:**
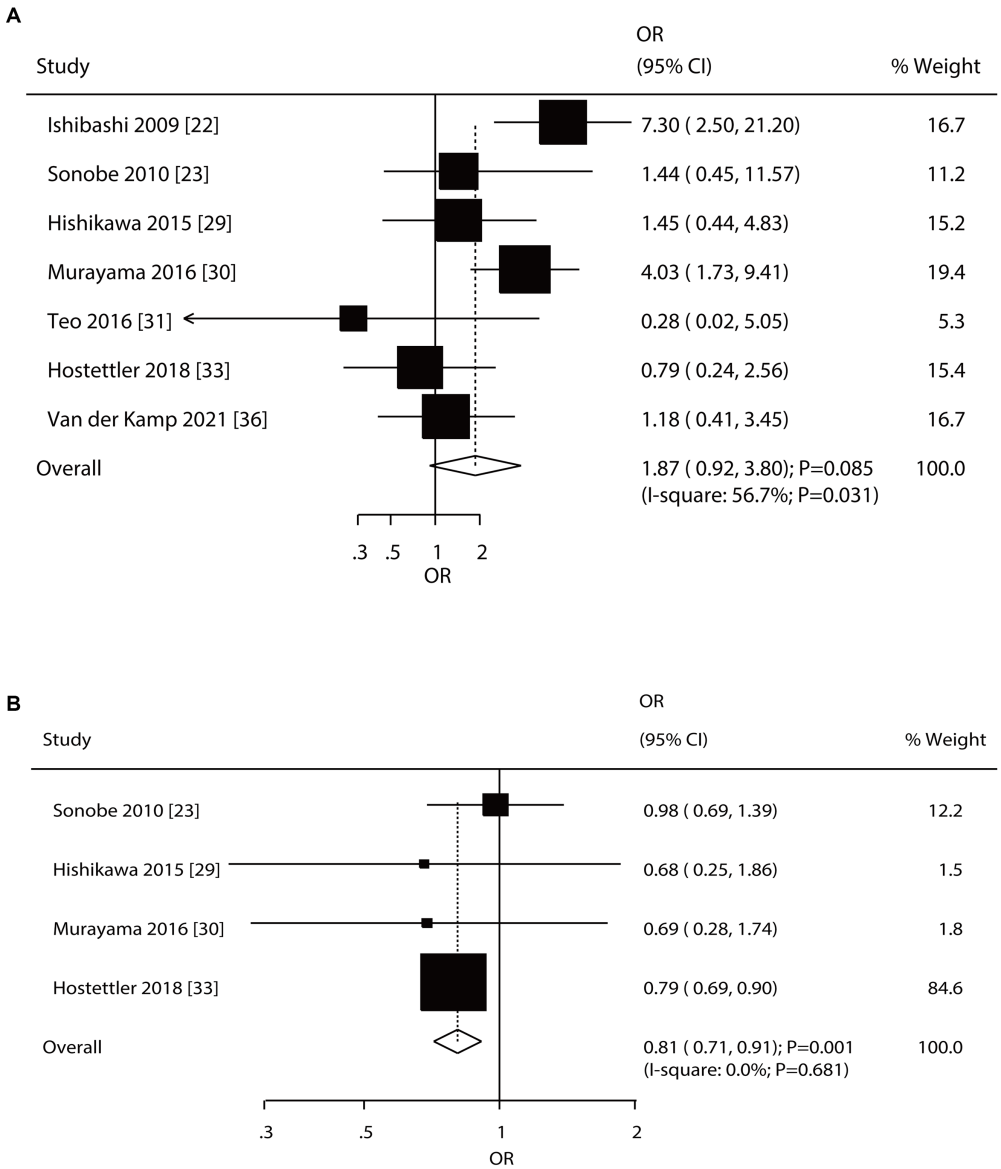
Effect of a history of subarachnoid hemorrhage (SAH) and a family history of SAH on the risk of aneurysm rupture in patients with unruptured intracranial aneurysms (UIA). **(A)** History of SAH. **(B)** Family history of SAH.

Seven and four studies reported the roles of a history of SAH and a family history of SAH on the risk of aneurysm rupture in patients with UIA, respectively (Figure 5). Notably, patients with a history of SAH were not associated with the risk of aneurysm rupture (OR: 1.87; 95% CI: 0.92–3.80; *p* = 0.085); in contrast, a family history of SAH was associated with a reduced risk of aneurysm rupture (OR: 0.81; 95% CI: 0.71–0.91; *p* = 0.001). There was significant heterogeneity in the history of SAH (*I*^2^ = 56.7%; *p* = 0.031); in contrast, there was no evidence of heterogeneity in the family history of SAH (*I*^2^ = 0.0%; *p* = 0.681). The pooled conclusions regarding the role of a history of SAH and a family history of SAH on the risk of aneurysm rupture were variable (Supplementary File 1). Subgroup analysis showed that patients with a history of SAH were associated with an increased risk of aneurysm rupture when pooled studies were conducted in Eastern countries, prospective cohort studies, a follow-up of <3.0 years, and high-quality studies, and the association between a history of SAH and aneurysm rupture risk was affected by region (*p* = 0.005), study design (*p* = 0.005), and follow-up (*p* = 0.021). Moreover, a family history of SAH was associated with a reduced risk of aneurysm rupture if pooled studies were conducted in Western countries, retrospective cohort studies, a follow-up of ≥3.0 years, and studies with high quality (Table 2). There was no significant publication bias for a history of SAH (*p*-value for Egger: 0.161; *p*-value for Begg: 0.548) or a family history of SAH (*p*-value for Egger: 0.940; *p*-value for Begg: 0.734; Supplementary File 2).

### 3.7 Aneurysm size and multiple aneurysms

**Figure 6:**
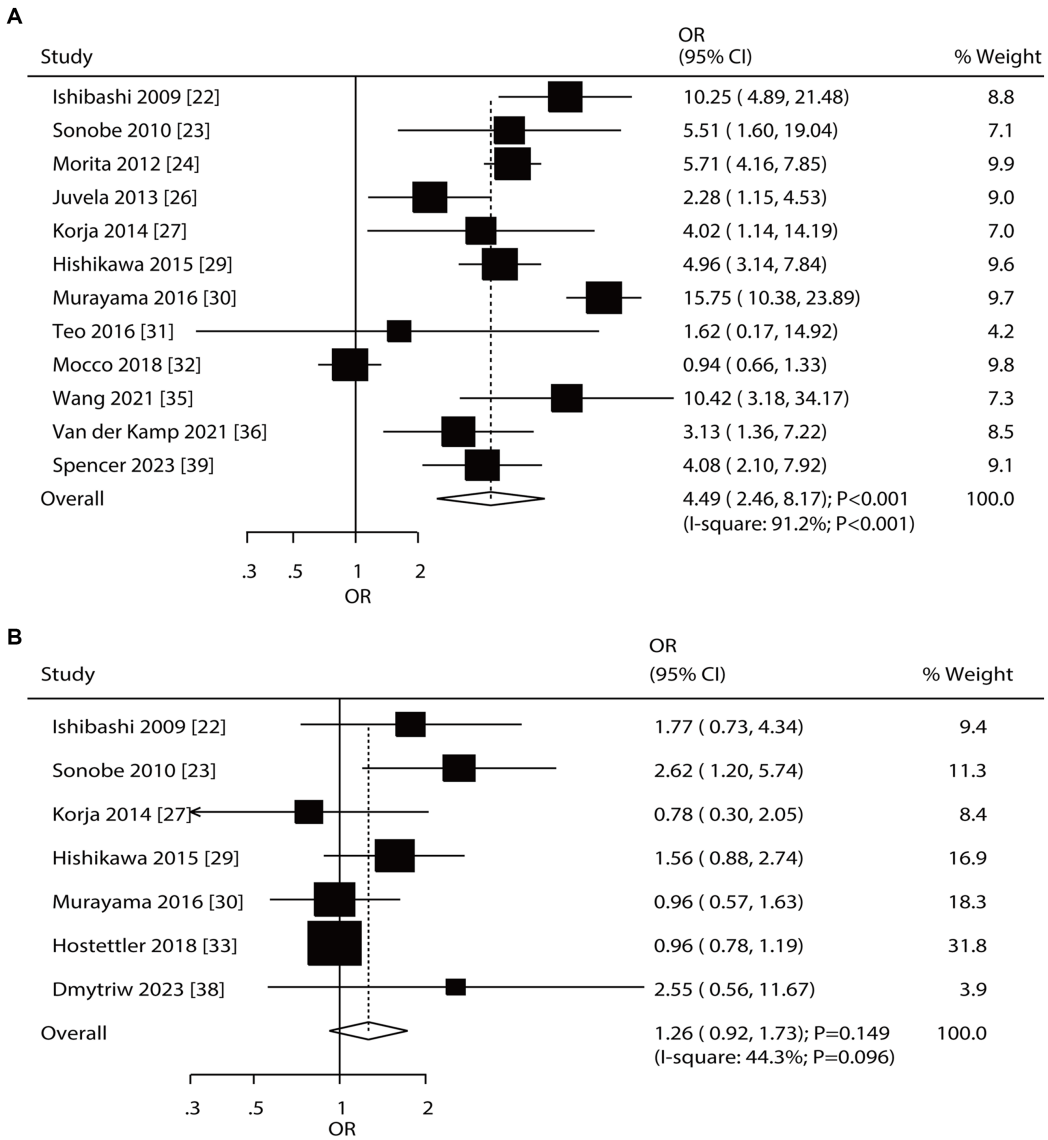
Effect of aneurysm size and multiple aneurysms on the risk of aneurysm rupture in patients with unruptured intracranial aneurysms (UIA). **(A)** Large size of aneurysm. **(B)** Multiple aneurysm.

Twelve and seven studies reported the roles of aneurysm size and multiple aneurysms on the risk of aneurysm rupture in patients with UIA, respectively (Figure 6). Large-size aneurysm was associated with an increased risk of aneurysm rupture (OR: 4.49; 95% CI: 2.46–8.17; *p* < 0.001); in contrast, multiple aneurysms were not associated with the risk of aneurysm rupture (OR: 1.26; 95% CI: 0.92–1.73; *p* = 0.149). There was significant heterogeneity in aneurysm size (*I*^2^ = 91.2%; *p* < 0.001) and multiple aneurysms (*I*^2^ = 44.3%; *p* = 0.096). The summary results for the role of aneurysm size and multiple aneurysms on the risk of aneurysm rupture were stable and were not altered by removing a single study (Supplementary File 1). Subgroup analyses showed that a large aneurysm size was associated with an increased risk of aneurysm rupture in most subgroups; in contrast, no significant association between a large-size aneurysm and the risk of aneurysm rupture was found in a moderate-quality pooled study. Moreover, the role of a large-size aneurysm in the risk of aneurysm rupture was affected by the region (*p* < 0.001) and follow-up (*p* < 0.001). Although the role of multiple aneurysms in the risk of aneurysm rupture was affected by the region (*p* = 0.041), none of the subgroups showed significant associations between multiple aneurysms and the risk of aneurysm rupture (Table 2). There was no significant publication bias for a large-size aneurysm (*p*-value for Egger: 0.784; *p*-value for Begg: 0.451) and multiple aneurysms (*p*-value for Egger: 0.099; *p*-value for Begg: 0.548; Supplementary File 2).

### 3.8 Aneurysm location

**Figure 7:**
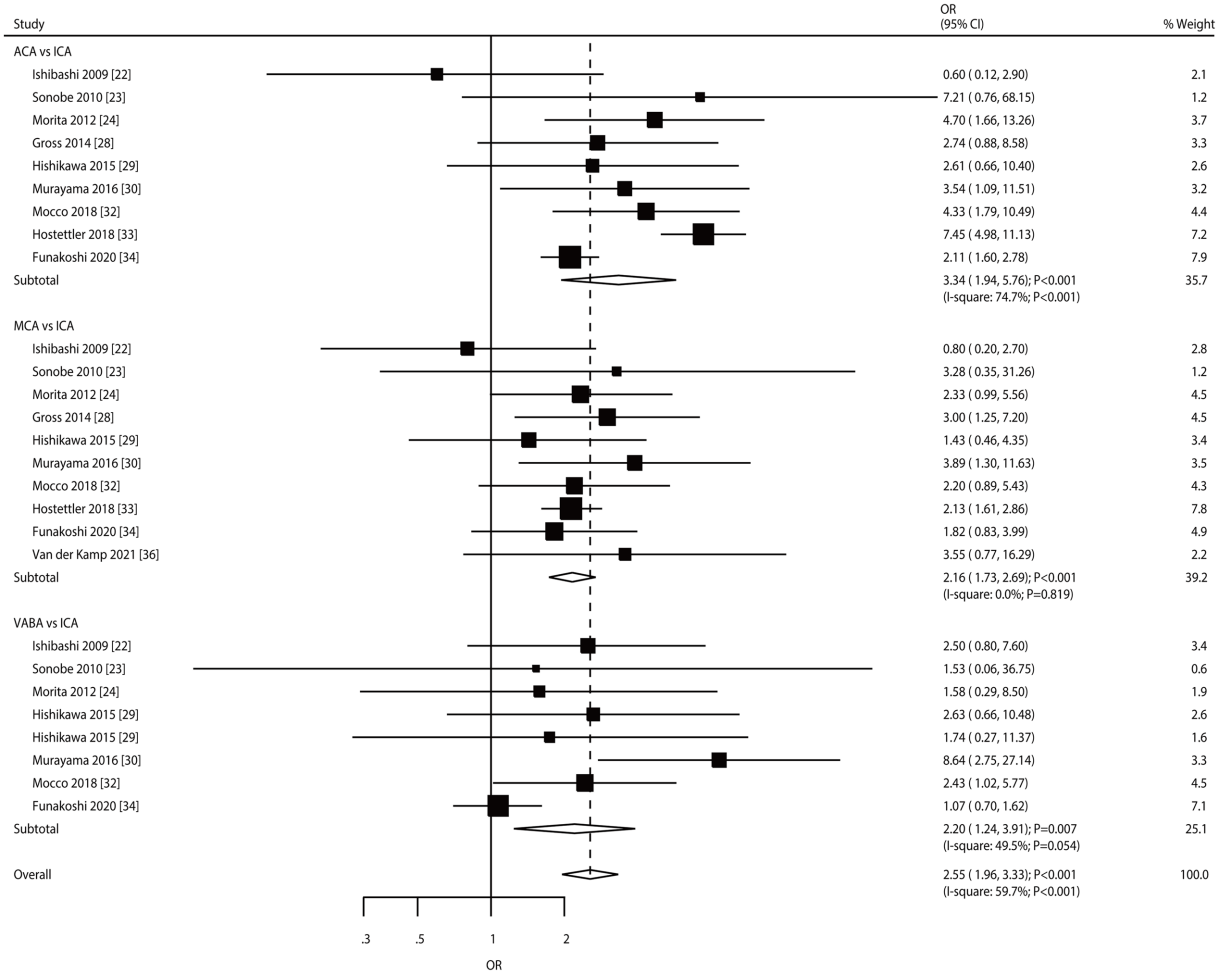
Effect of aneurysm location on the risk of aneurysm rupture in patients with unruptured intracranial aneurysms (UIA).

Nine, ten, and eight studies reported the roles of the ACA, MCA, and VABA versus the ICA, respectively, in the risk of aneurysm rupture in patients with UIA (Figure 7). ACA (OR: 3.34; 95% CI: 1.94–5.76; *p* < 0.001), MCA (OR: 2.16; 95% CI: 1.73–2.69; *p* < 0.001), and VABA (OR: 2.20; 95% CI: 1.24–3.91; *p* = 0.007) were associated with an increased risk of aneurysm rupture. There was significant heterogeneity in ACA (*I*^2^ = 74.7%; *p* < 0.001) and VABA (*I*^2^ = 49.5%; *p* = 0.054); in contrast, there was no heterogeneity in the MCA (*I*^2^ = 0.0%; *p* = 0.819). Sensitivity analyses indicated that the pooled conclusions regarding the roles of ACA, MCA, and VABA in the risk of aneurysm rupture were stable (Supplementary File 1). The subgroup analyses indicated that the roles of ACA and MCA in the risk of aneurysm rupture were consistent with the overall analysis of all subgroups. VABA was not associated with the risk of aneurysm rupture when pooled studies were designed as retrospective cohorts, a follow-up of ≥3.0 years, and high-quality studies (Table 2). There was no significant publication bias for ACA (*p*-value for Egger: 0.811; *p*-value for Begg: 0.602), MCA (*p*-value for Egger: 0.845; *p*-value for Begg: 0.858), or VABA (*p*-value for Egger: 0.146; *p*-value for Begg: 0.386; Supplementary File 2).

## 4 Discussion

The predictors of aneurysm rupture in patients with UIA should be further identified to screen patients at high risk of aneurysm rupture. Here, we performed a large quantitative study to identify 17,069 patients with UIA and 2,699 aneurysm ruptures in 18 studies and reviewed the characteristics of studies or patients across a broad range. We determined that large aneurysms, ACA, MCA, and VABA were associated with an increased risk of aneurysm rupture; in contrast, hyperlipidemia and a family history of SAH played a protective role in the risk of aneurysm rupture. Furthermore, age, sex, current smoking status, hypertension, DM, a history of SAH, and multiple aneurysms were not associated with the risk of aneurysm rupture. Finally, the region, study design, follow-up, and study quality could predict the risk of aneurysm rupture in patients with UIA.

Several meta-analyses have investigated potential predictors of aneurysm rupture risk in patients with UIA (5, 9–11). Greving et al. (5) identified six prospective studies and found that the predictors of aneurysm rupture included age, hypertension, a history of SAH, aneurysm size, aneurysm location, and geographic region. Han et al. (9) identified 15 studies and found that wall shear stress, oscillatory shear index, and low shear index could affect the risk of aneurysm rupture in patients. Shu et al. (10) identified four studies reporting machine learning algorithms for rupture risk in patients with UIA and found that the diagnostic value of machine learning algorithms was excellent, with sensitivity and specificity of 84% and 78%, respectively. Guo et al. (11) identified eight studies and found that aspirin plays a protective role against the risk of growth and rupture of aneurysms in patients with UIA. However, these studies did not perform exploratory analyses, and the predictors of aneurysm rupture in patients with UIA should be further explored.

The study indicates that age and sex were not associated with aneurysm rupture risk in patients with UIA. However, subgroup analyses showed that younger age was associated with a lower risk of aneurysm rupture when the follow-up was <3.0 years, inconsistent with the findings of a prior meta-analysis (5). This discrepancy could be explained by variations in the reference age group, which may have affected the estimated effect of age on the risk of aneurysm rupture. Moreover, female patients were associated with an increased risk of aneurysm rupture as compared with male patients when pooled from prospective cohort studies and a follow-up of <3.0 years; this might be due to the higher prevalence of UIA in women compared to men and the accelerated growth rate in women, which was associated with an increased risk of aneurysm rupture (40). Additionally, the risk of aneurysm rupture was not affected by smoking status, hypertension, and DM. Exploratory analysis revealed that current smoking was associated with an increased risk of aneurysm rupture when pooled studies were conducted in Western countries, studies with retrospective cohorts, and a follow-up of ≥3.0 years. This observation could be because smoking is associated with an acute increase in blood pressure for nearly 3 h, and this transient increase might play an important role in the risk of aneurysm rupture (41). Moreover, long-term smoking can change the formation of aneurysms by weakening the vessel walls of cerebral arteries (42). Hypertension was associated with an increased risk of aneurysm rupture in pooled prospective cohort studies, inconsistent with a previous meta-analysis (5), which could be explained by the use of antihypertensive agents, which is associated with a reduced risk of aneurysm rupture (43). Finally, the subgroup analyses showed that DM plays a protective role in the risk of aneurysm rupture when pooled with high-quality studies, which might be affected by hypoglycemic drugs in patients with DM.

This study showed that hyperlipidemia was associated with a reduced risk of aneurysm rupture, which could be explained by the use of statins that reduce the risk of aneurysm rupture through lipid-lowering effects, anti-inflammation of the vasculature, and the ability to stimulate ECM production of extracellular matrix (44–46). Moreover, a history of SAH was not associated with the risk of aneurysm rupture, indicating that aneurysm rupture did not interact with other aneurysms in patients with multiple aneurysms. Notably, we determined that a family history of SAH was associated with a reduced risk of aneurysm rupture, which could be explained by careful monitoring to prevent rupture. Furthermore, the risk of aneurysm rupture can be affected by the size and location of the aneurysm, consistent with prior meta-analyses (5).

This study has some limitations. First, both prospective and retrospective cohort studies were included, and the results may have been affected by selection and recall biases. Second, the reference groups for age and aneurysm size differed across the included studies, which might have affected the estimates for these predictors. Third, the analyses included both crude data and adjusted results, and the adjusted variables might have affected the risk of aneurysm rupture. Fourth, the risk of aneurysm rupture differed according to the location and morphology of aneurysms. Fifth, the heterogeneity among the included studies was not fully explained by sensitivity and subgroup analyses, which could be explained by the different disease statuses of UIA. Finally, there was inevitable publication bias and a restricted detailed meta-analysis of published articles.

This study showed that the predictors of aneurysm rupture in patients with UIA included hyperlipidemia, a family history of SAH, a large-size aneurysm, ACA, MCA, and VABA. However, age, sex, smoking status, hypertension, DM, a history of SAH, and multiple aneurysms did not affect the risk of aneurysm rupture in patients with UIA. The roles of these predictors for the aneurysm rupture risk could be affected by the region, study design, follow-up, and study quality.

## Data availability statement

The original contributions presented in the study are included in the article/Supplementary material, further inquiries can be directed to the corresponding author.

## Ethics statement

All analyses were based on previous published studies, thus no ethical approval and patient consent are required.

## Author contributions

JM: Conceptualization, Data curation, Formal analysis, Writing – original draft. YZ: Conceptualization, Data curation, Formal analysis, Writing – original draft. PL: Data curation, Formal analysis, Writing – original draft. TZ: Data curation, Formal analysis, Writing – original draft. ZS: Data curation, Formal analysis, Writing – original draft. TJ: Data curation, Formal analysis, Writing – original draft. AL: Conceptualization, Formal analysis, Project administration, Writing – review & editing.
