# Supplementary material for: Risk factors for the rupture of intracranial aneurysms: a systematic review and meta-analysis
Source: Front Neurol. 2023 Dec 11;14:1268438. doi: 10.3389/fneur.2023.1268438 (PMC10749344; doi:10.3389/fneur.2023.1268438)
Supplement: Supplementary file 1 [file Data_Sheet_1.DOCX]

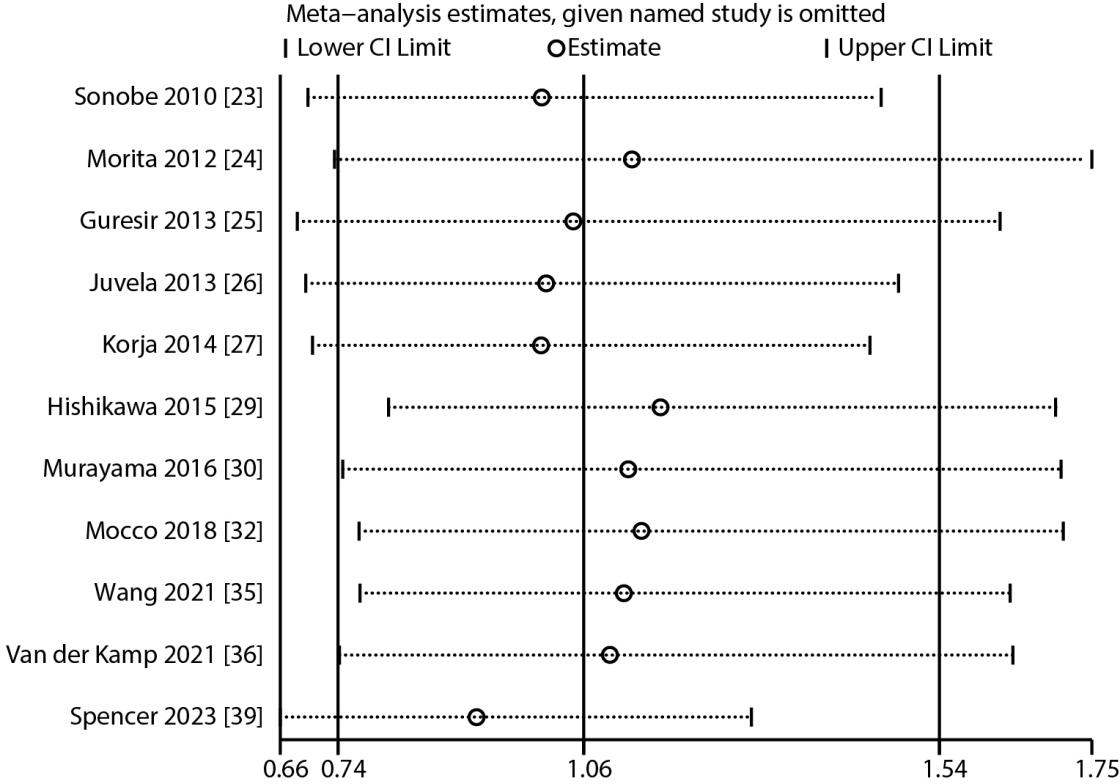


Figure S1. Sensitivity analysis for age (younger vs elder) on the risk of aneurysm rupture in UIA patients


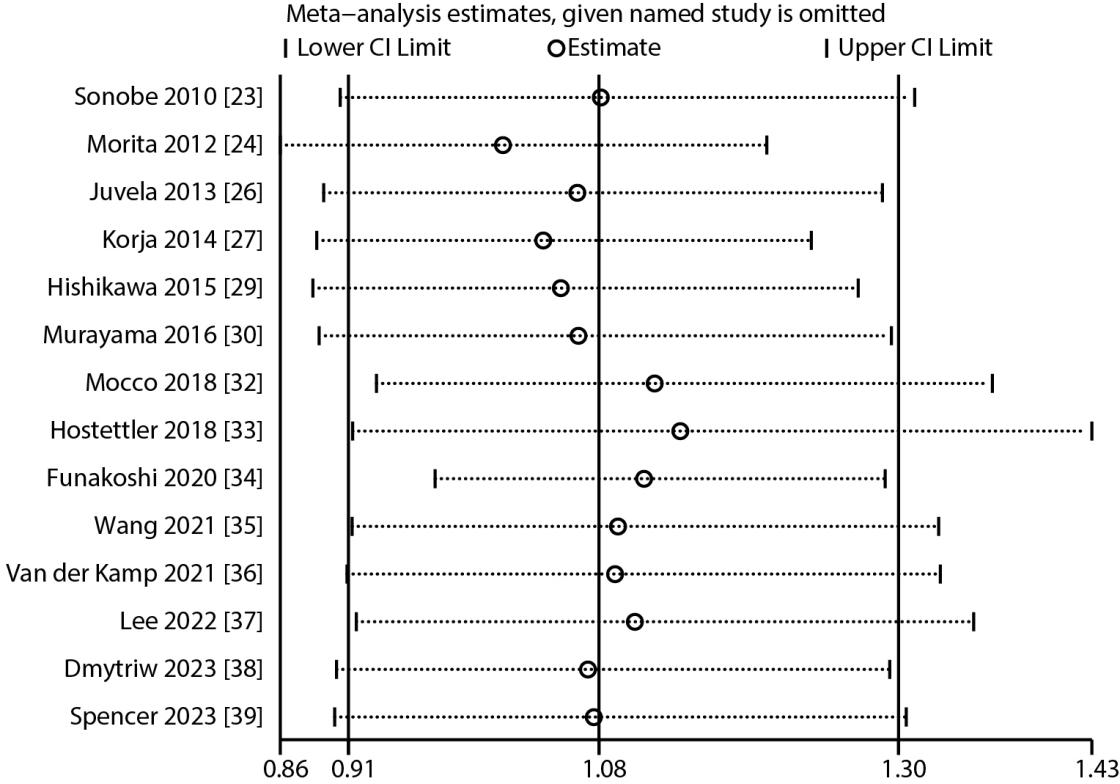


Figure S2. Sensitivity analysis for gender (female vs male) on the risk of aneurysm rupture in UIA patients


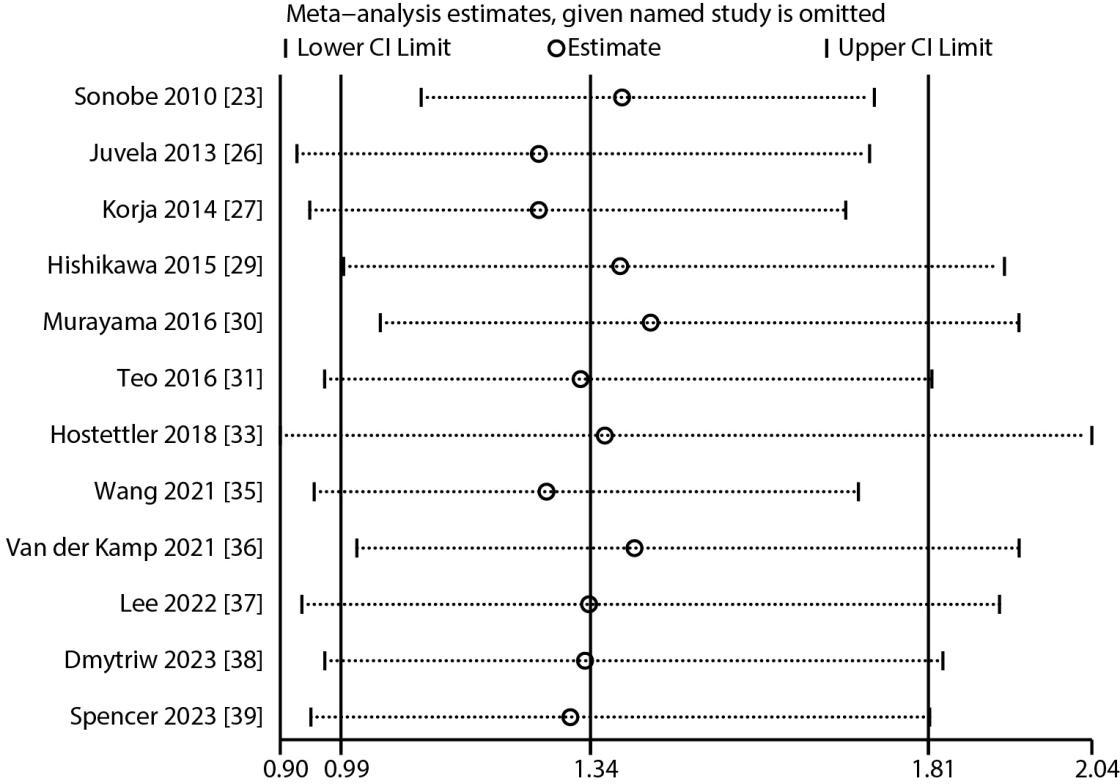


Figure S3. Sensitivity analysis for current smoker on the risk of aneurysm rupture in UIA patients


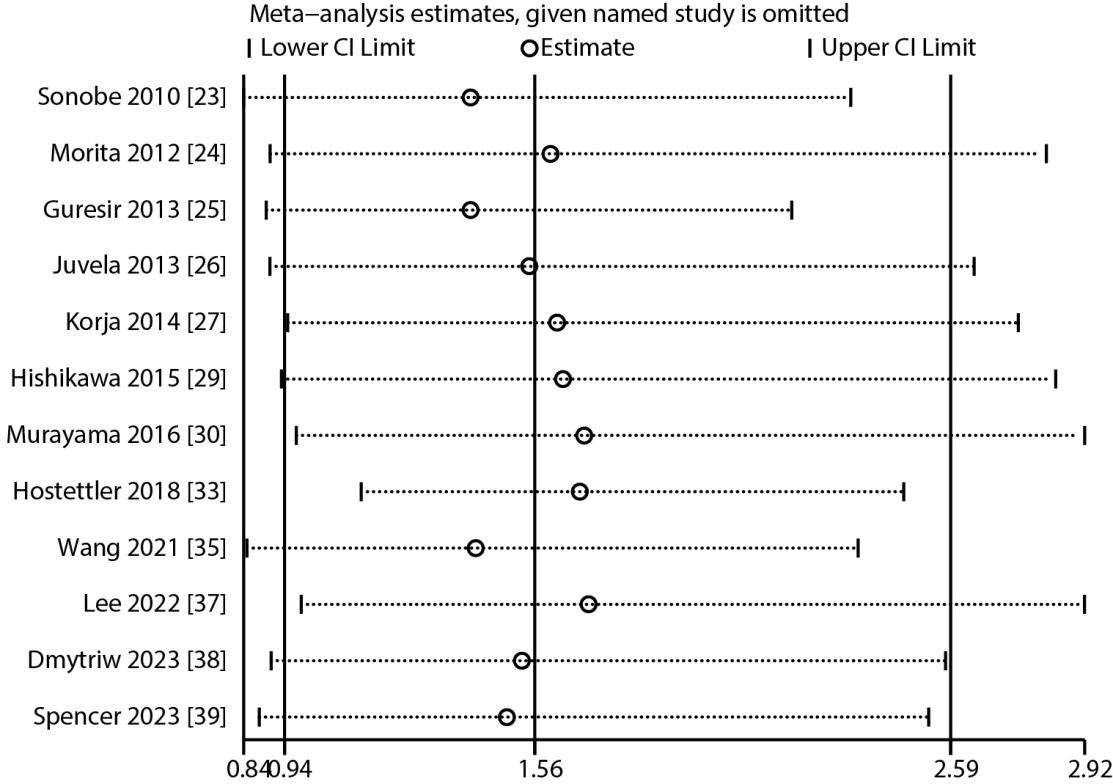


Figure S4. Sensitivity analysis for hypertension on the risk of aneurysm rupture in UIA patients


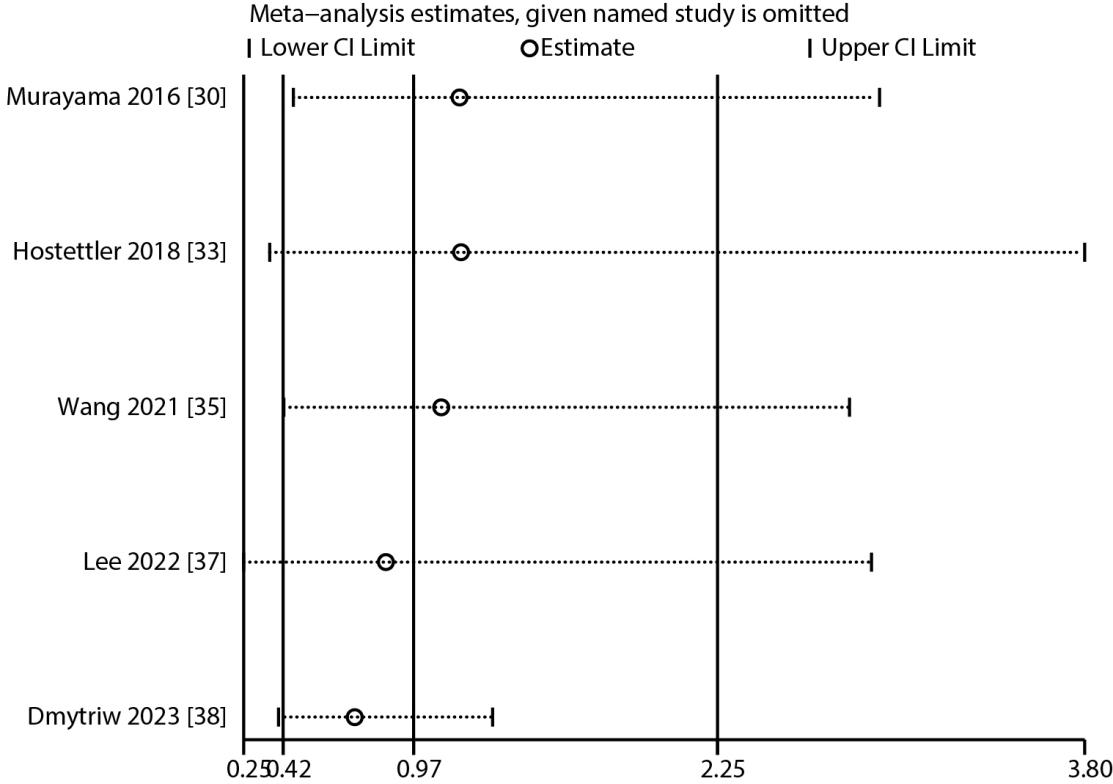


Figure S5. Sensitivity analysis for DM on the risk of aneurysm rupture in UIA patients


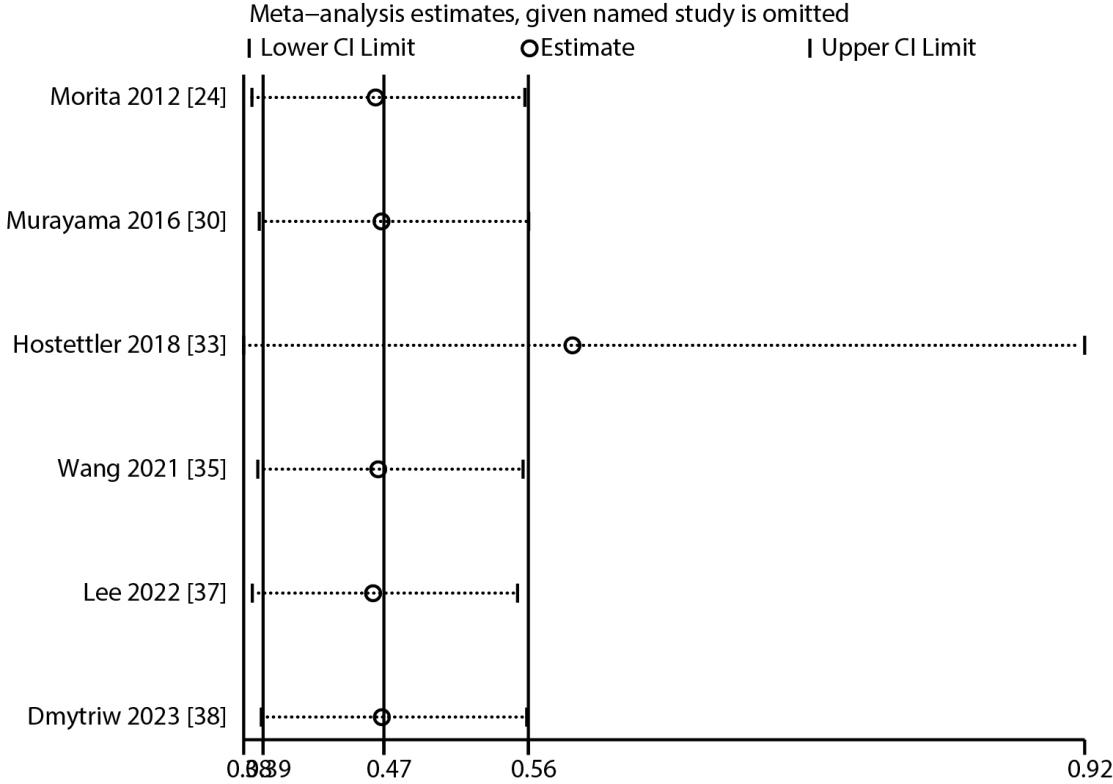


Figure S6. Sensitivity analysis for hyperlipidemia on the risk of aneurysm rupture in UIA patients


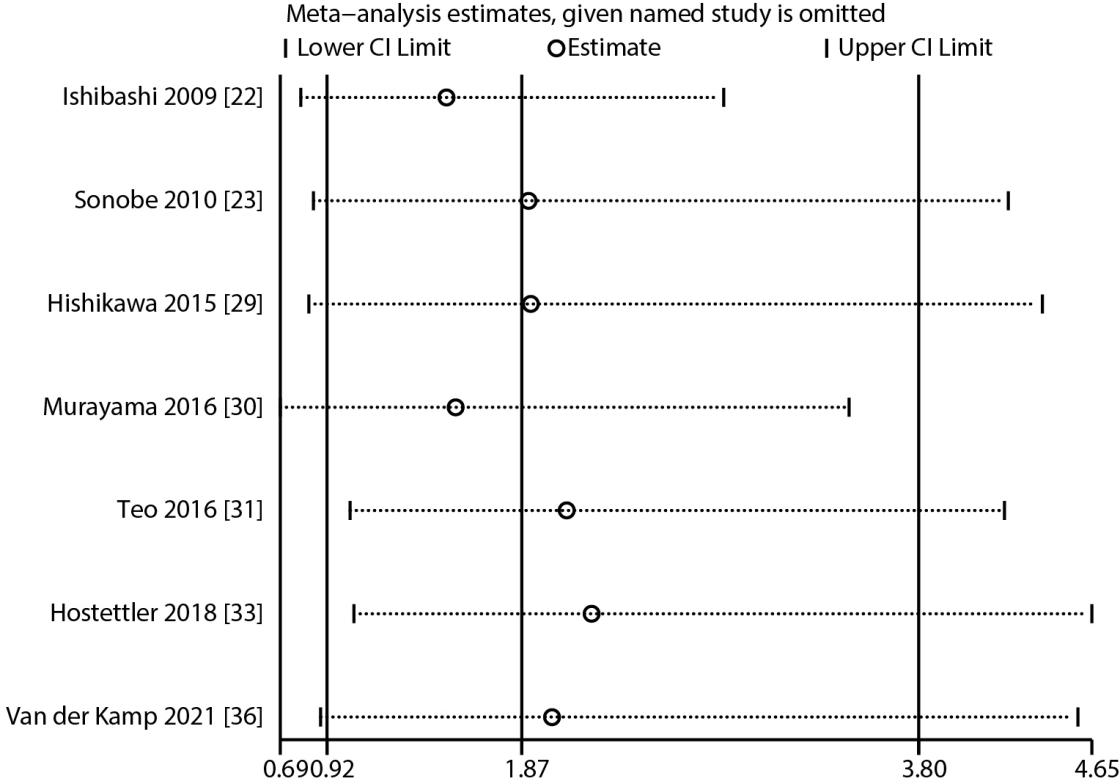


Figure S7. Sensitivity analysis for history of SAH on the risk of aneurysm rupture in UIA patients


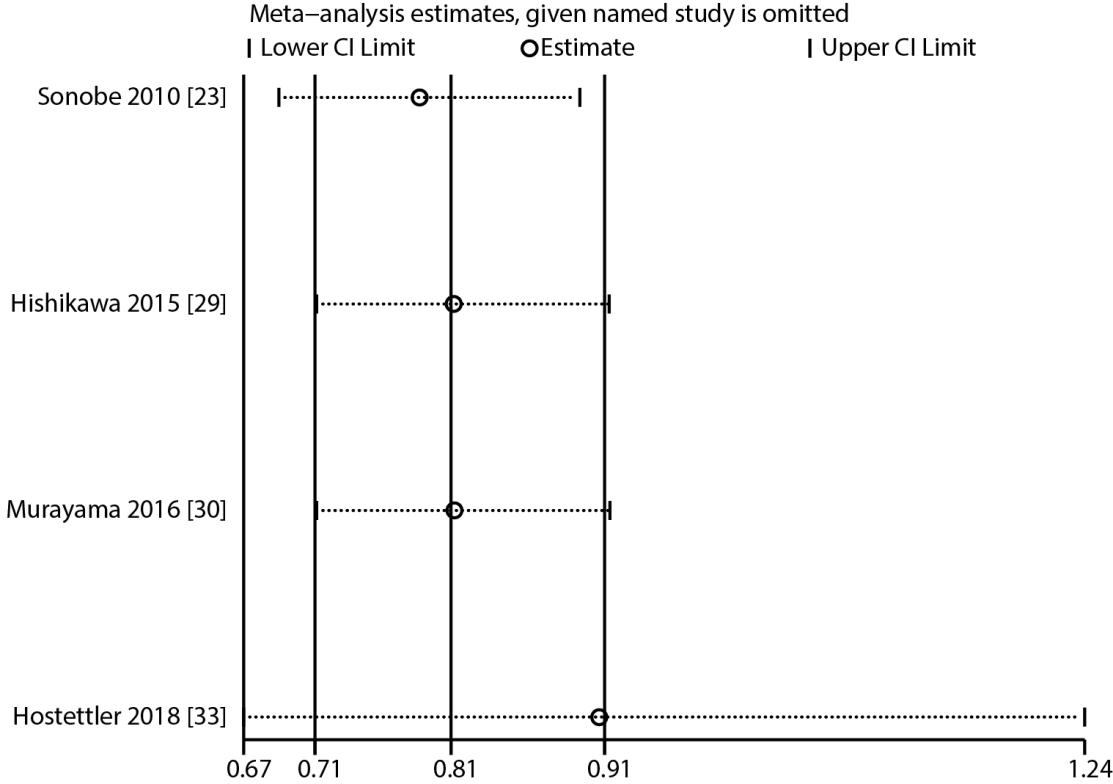


Figure S8. Sensitivity analysis for family history of SAH on the risk of aneurysm rupture in UIA patients


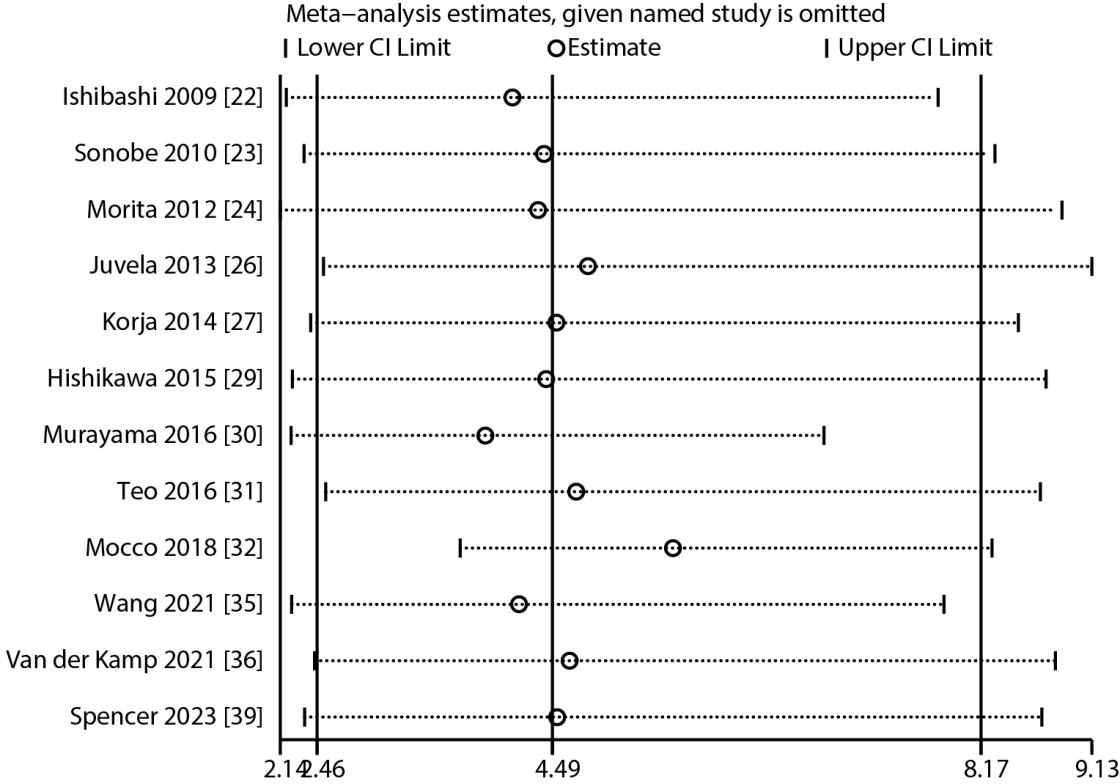


Figure S9. Sensitivity analysis for large size of aneurysm on the risk of aneurysm rupture in UIA patients


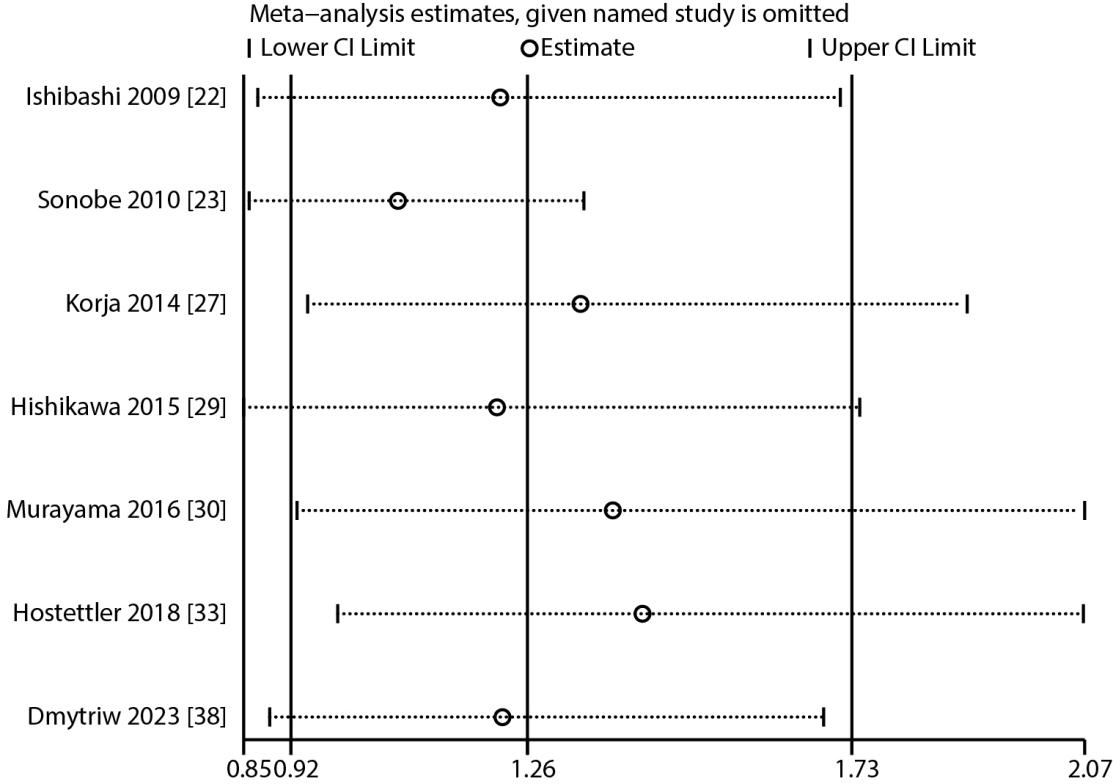


Figure S10. Sensitivity analysis for multiple aneurysm on the risk of aneurysm rupture in UIA patients


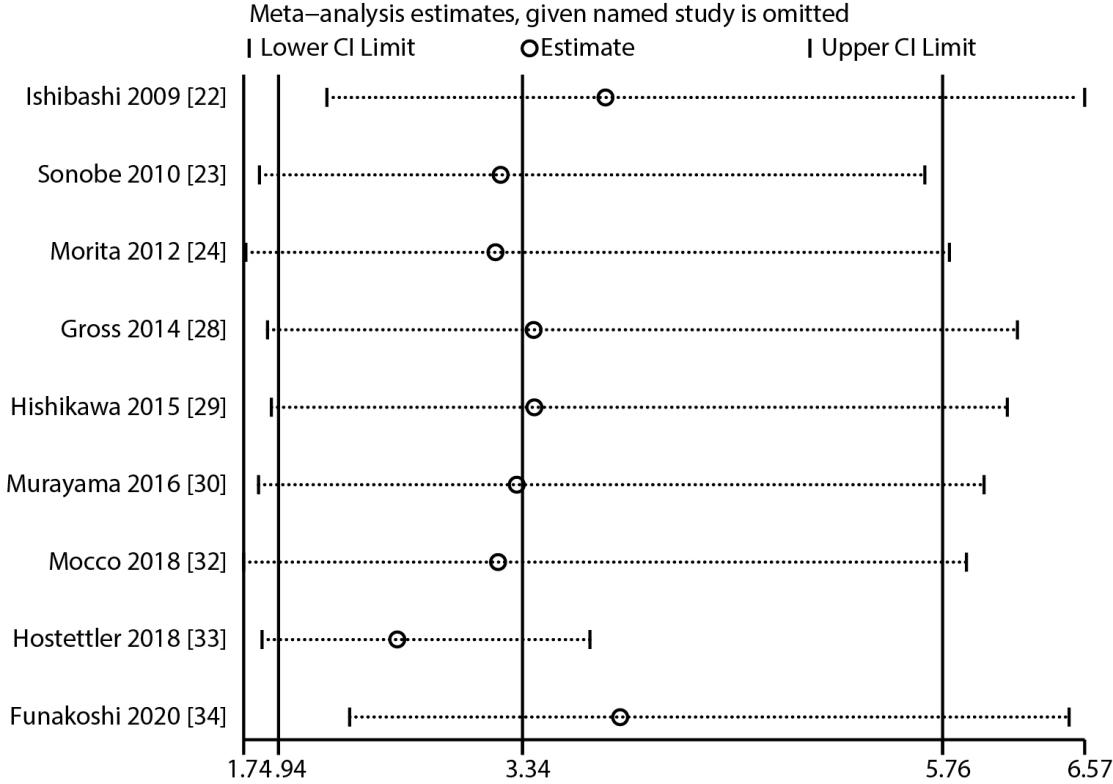


Figure S11. Sensitivity analysis for ACA vs ICA on the risk of aneurysm rupture in UIA patients


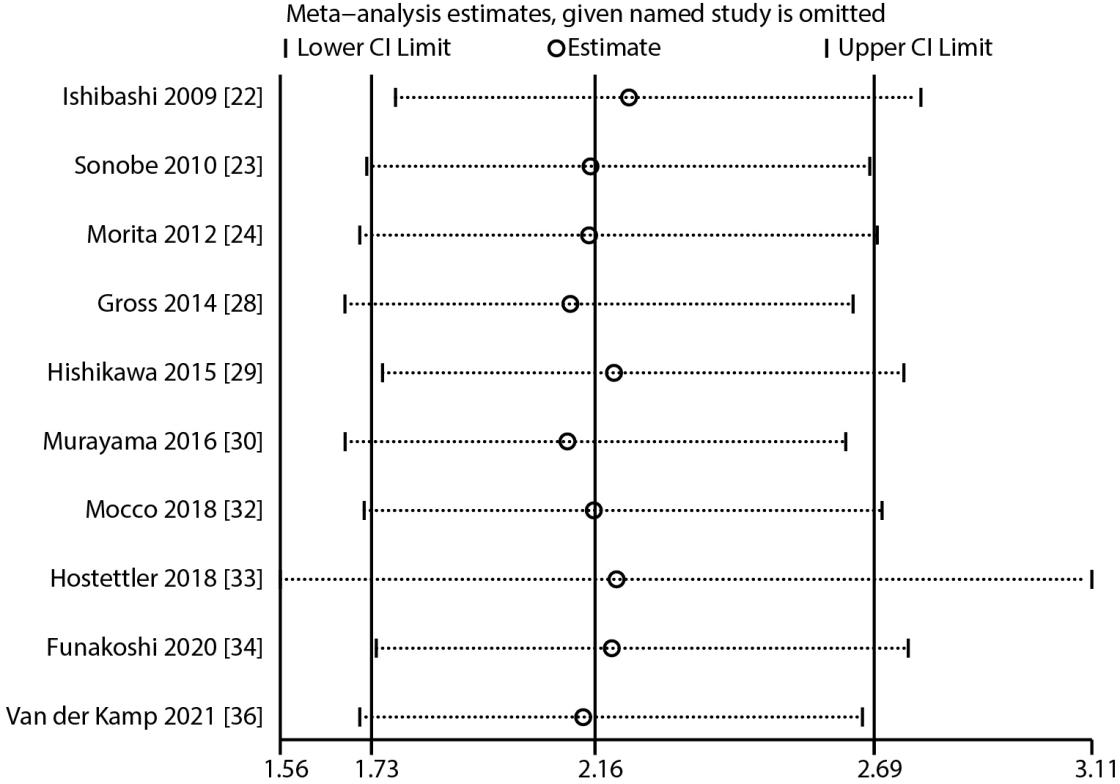


Figure S12. Sensitivity analysis for MCA vs ICA on the risk of aneurysm rupture in UIA patients


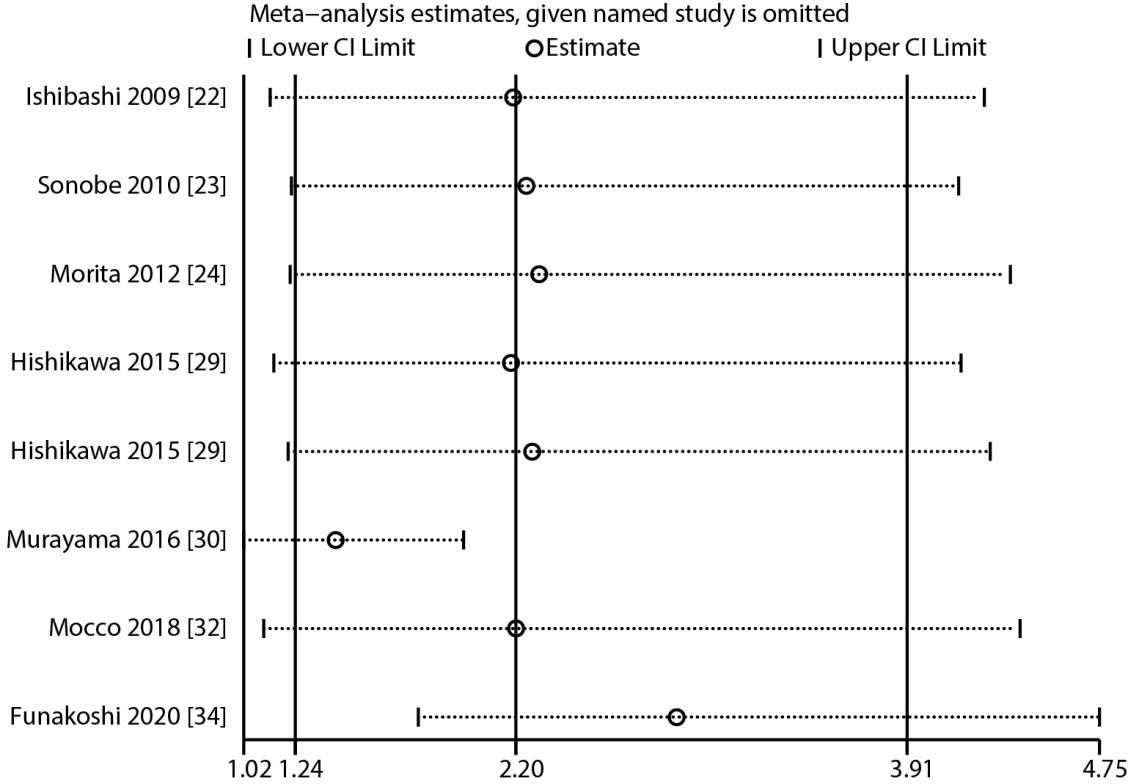


Figure S13. Sensitivity analysis for VABA vs ICA on the risk of aneurysm rupture in UIA patients
