# Supplementary material for: Risk factors for the rupture of intracranial aneurysms: a systematic review and meta-analysis
Source: Front Neurol. 2023 Dec 11;14:1268438. doi: 10.3389/fneur.2023.1268438 (PMC10749344; doi:10.3389/fneur.2023.1268438)
Supplement: Supplementary file 2 [file Data_Sheet_2.DOCX]

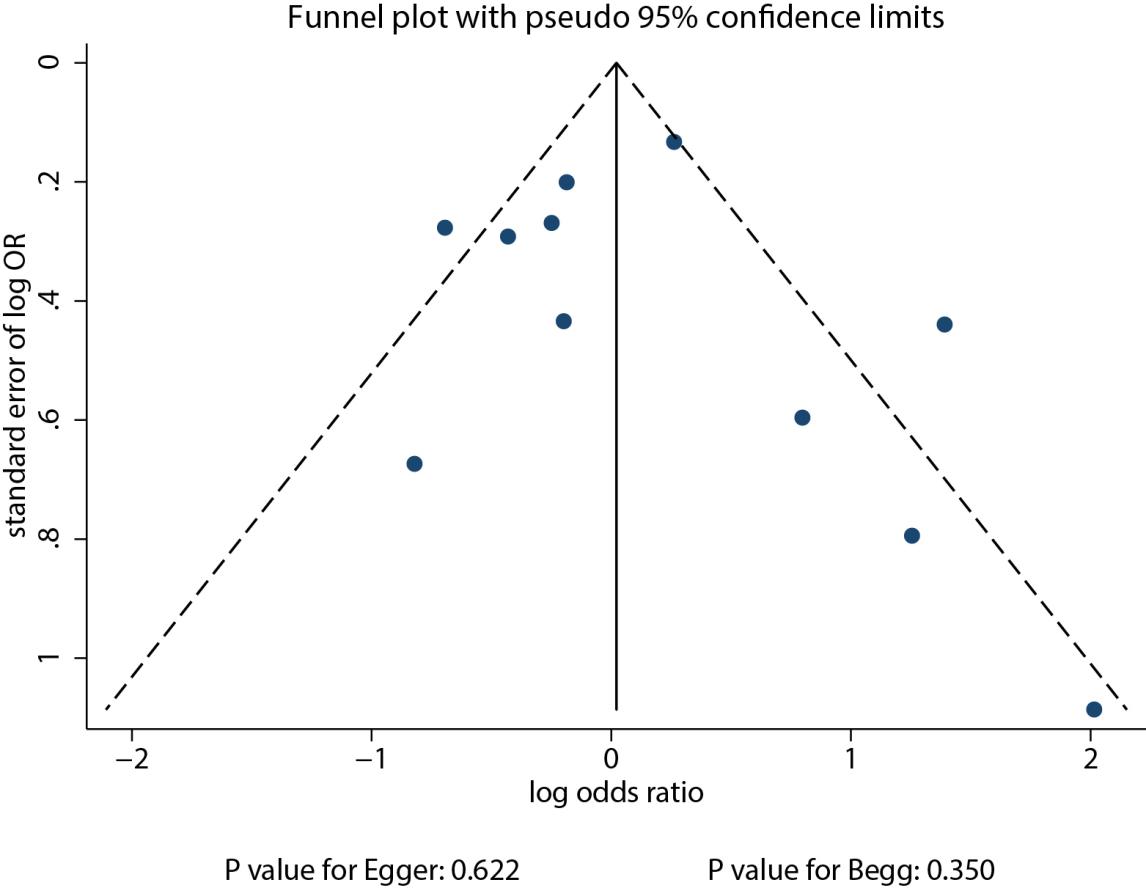


Figure S1. Funnel plot for age (younger vs elder) on the risk of aneurysm rupture in UIA patients


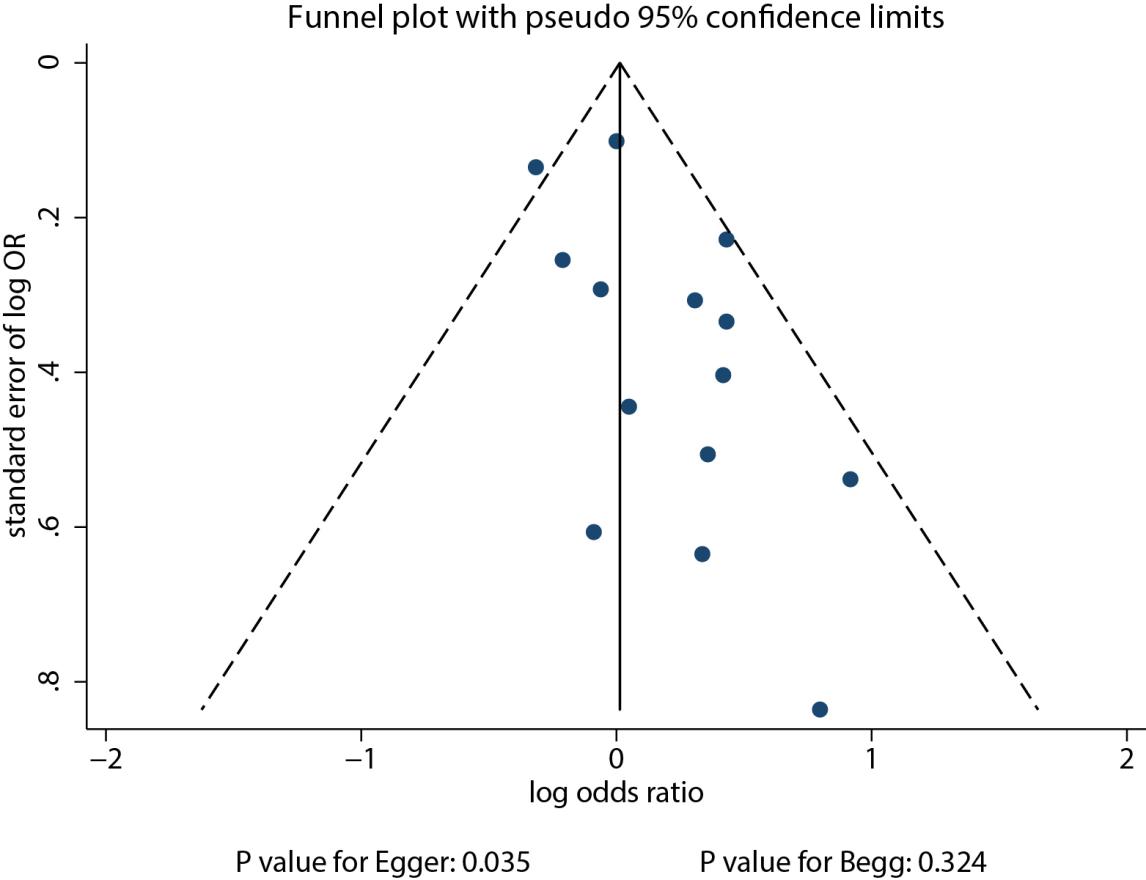


Figure S2. Funnel plot for gender (female vs male) on the risk of aneurysm rupture in UIA patients


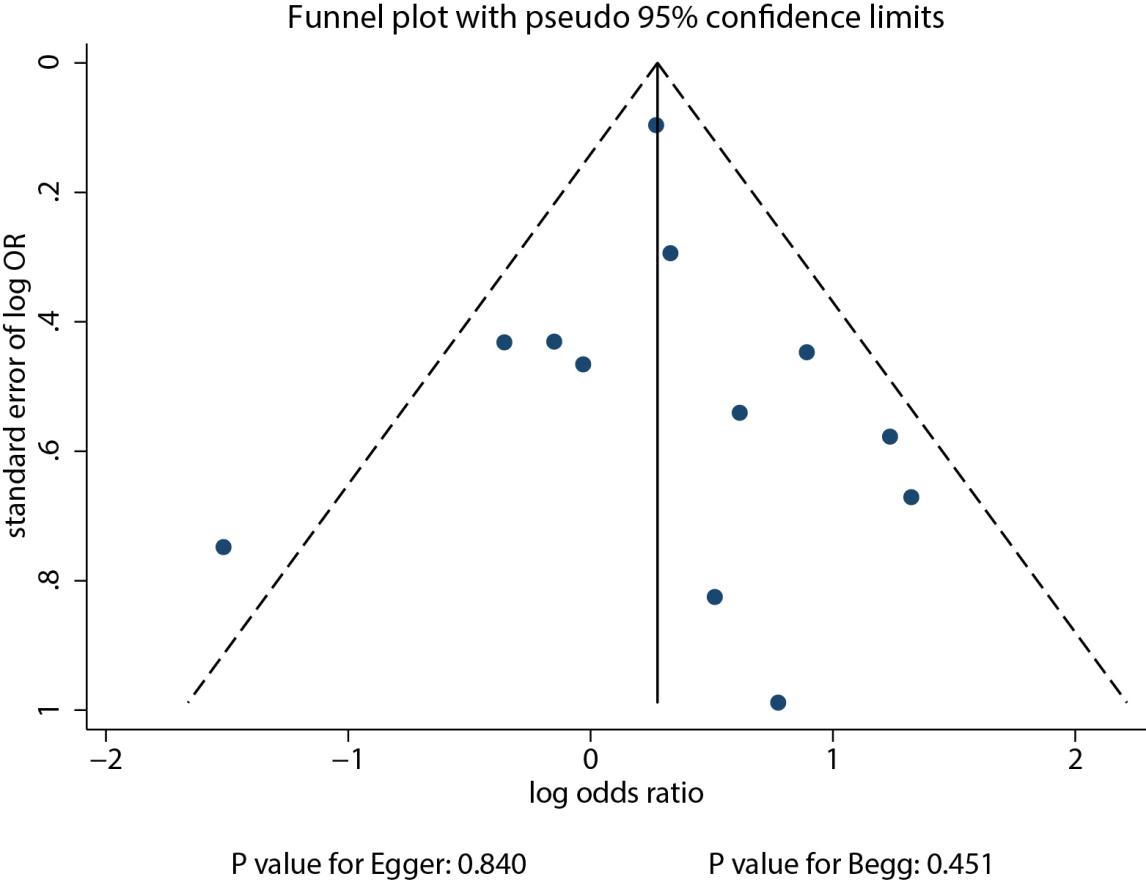


Figure S3. Funnel plot for current smoker on the risk of aneurysm rupture in UIA patients


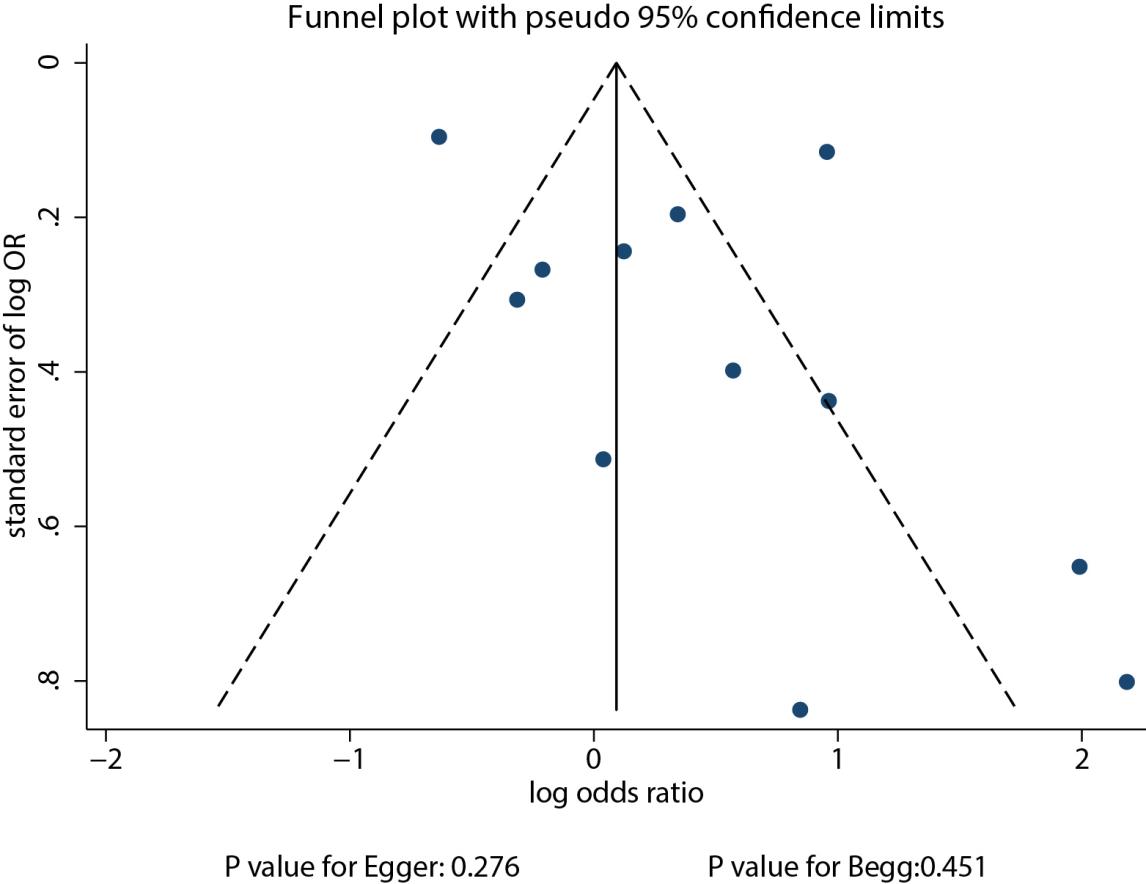


Figure S4. Funnel plot for hypertension on the risk of aneurysm rupture in UIA patients


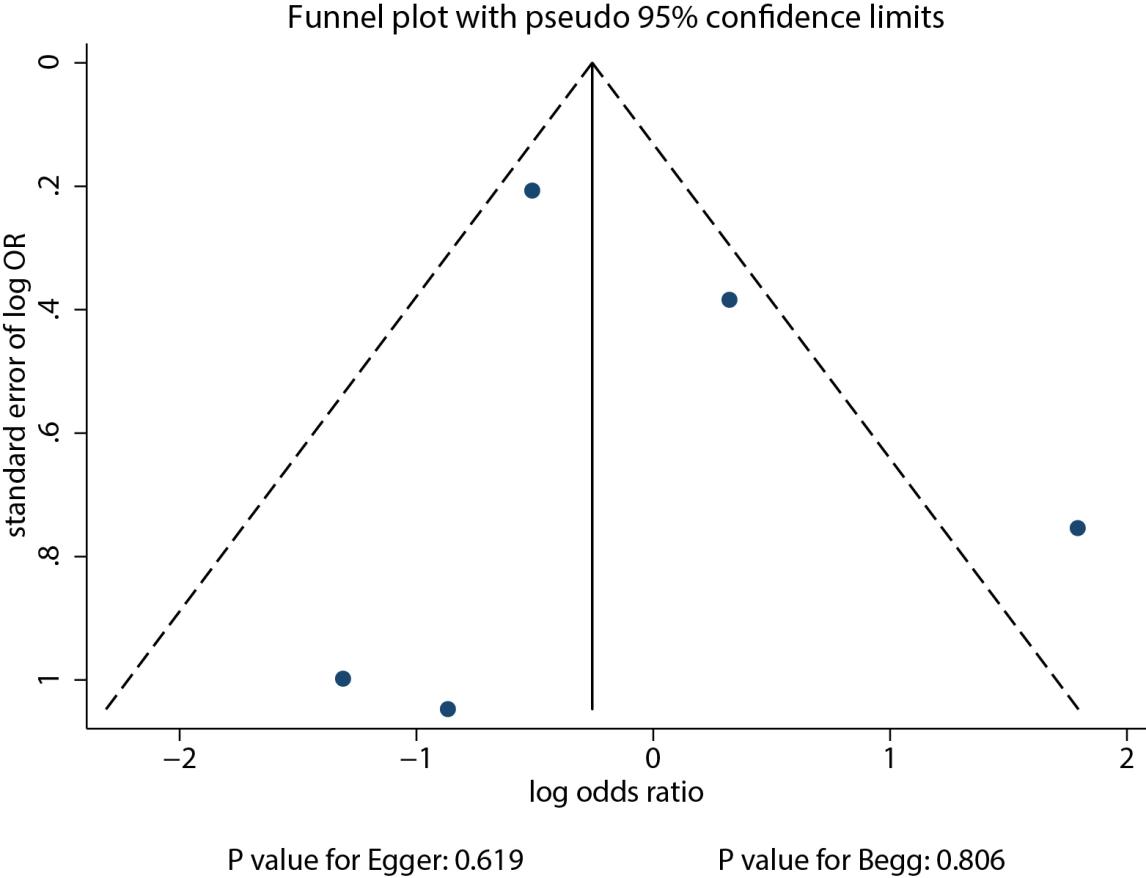


Figure S5. Funnel plot for DM on the risk of aneurysm rupture in UIA patients


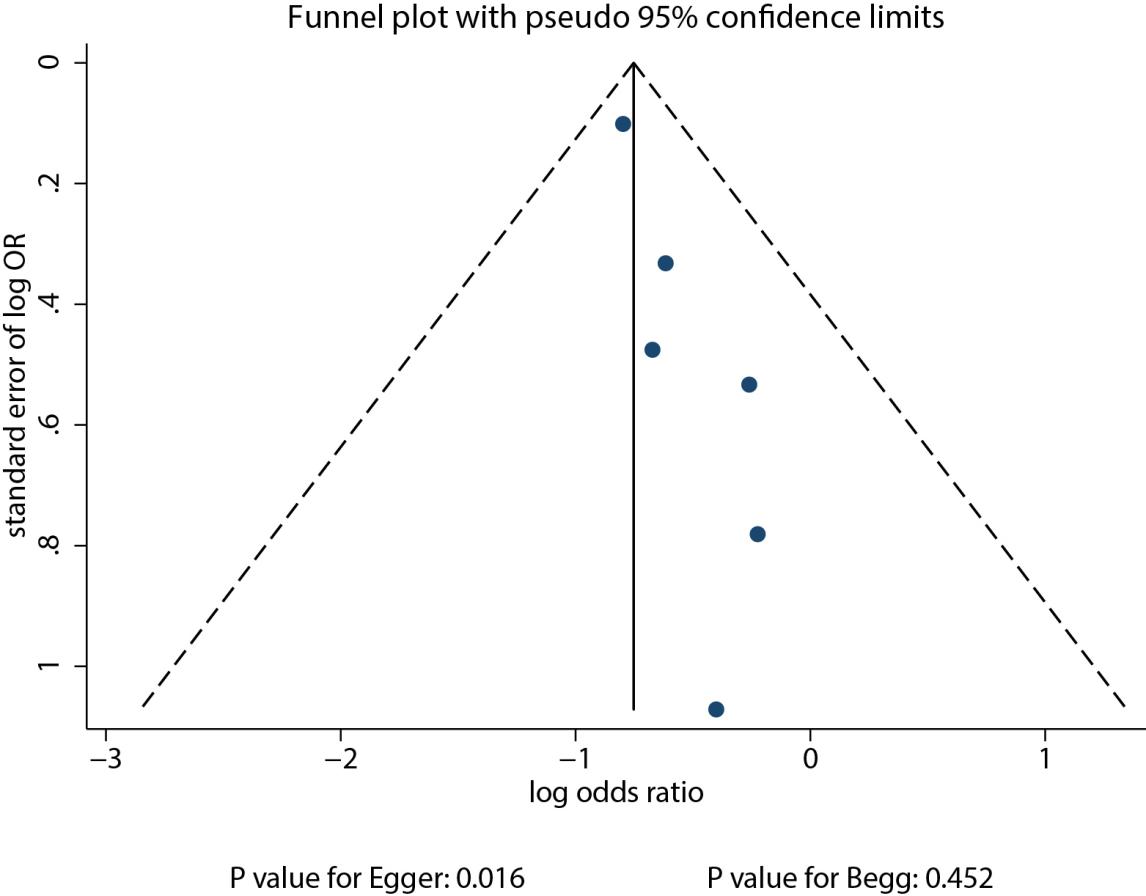


Figure S6. Funnel plot for hyperlipidemia on the risk of aneurysm rupture in UIA patients


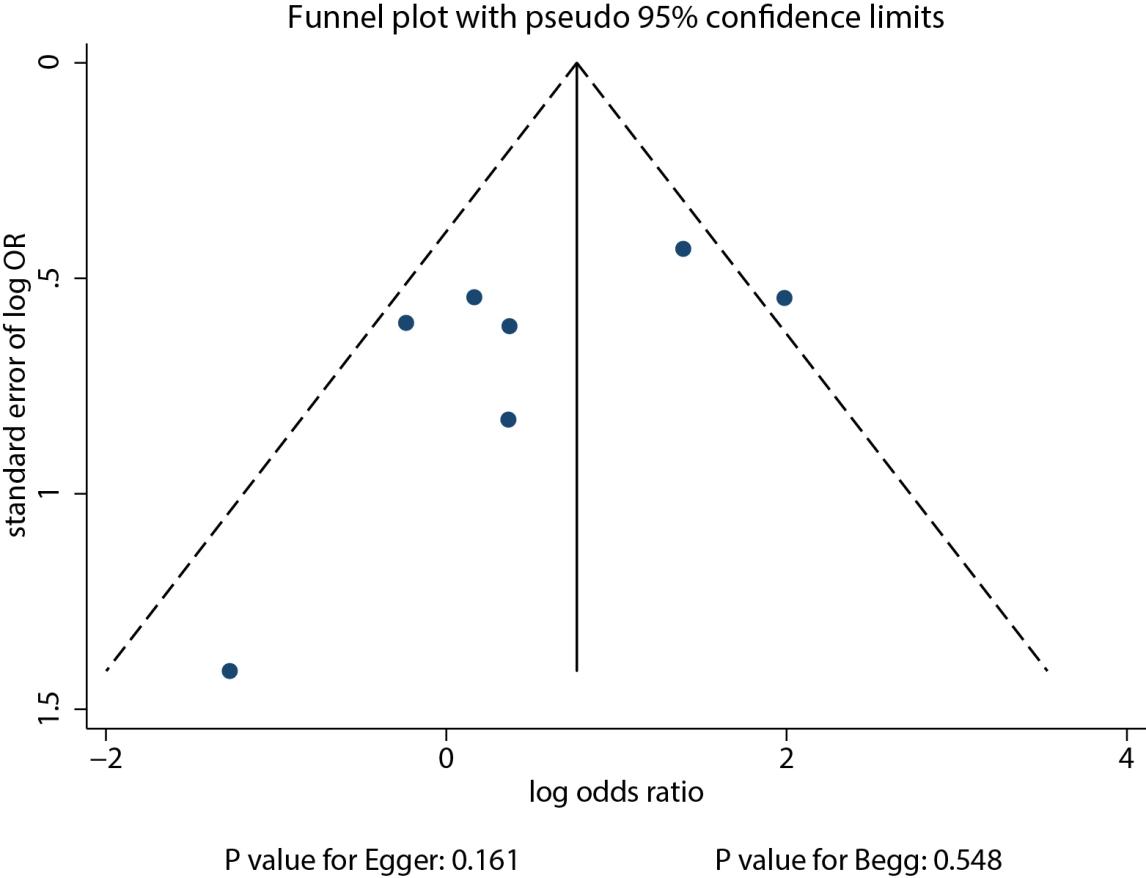


Figure S7. Funnel plot for history of SAH on the risk of aneurysm rupture in UIA patients


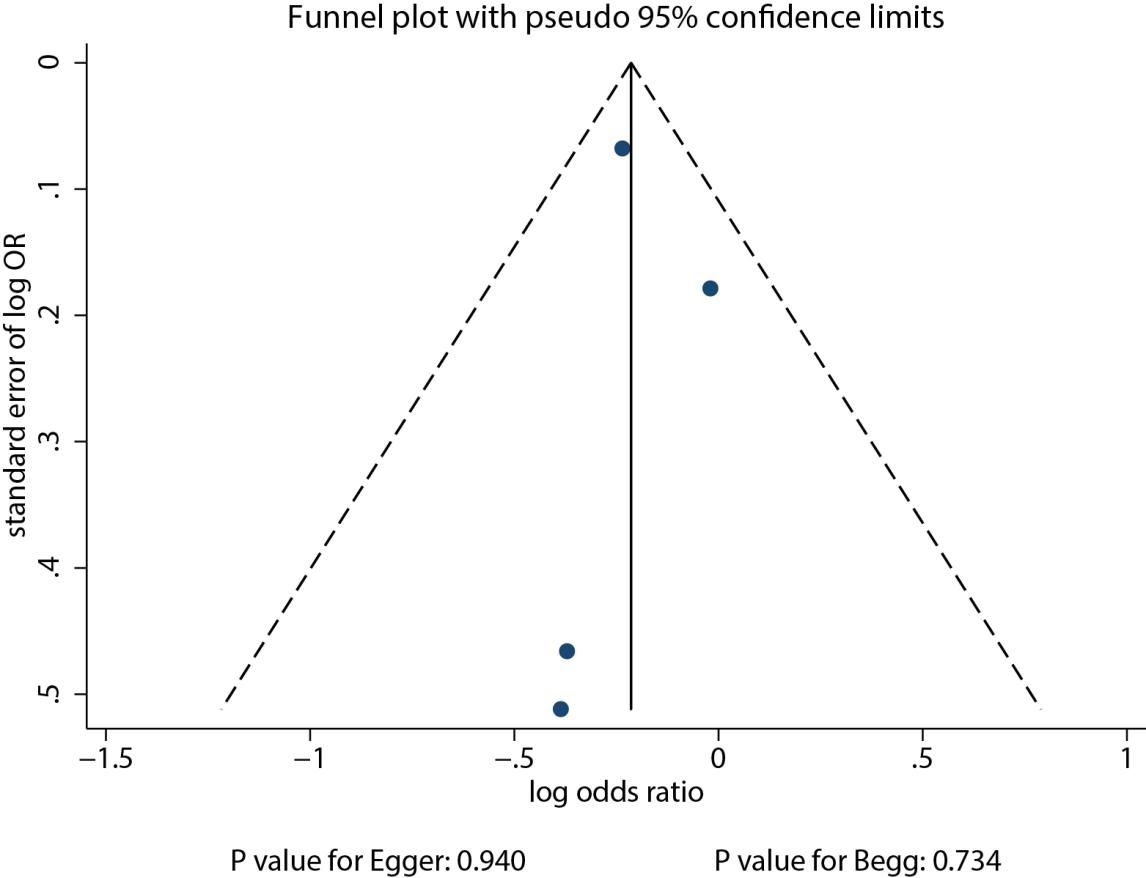


Figure S8. Funnel plot for family history of SAH on the risk of aneurysm rupture in UIA patients


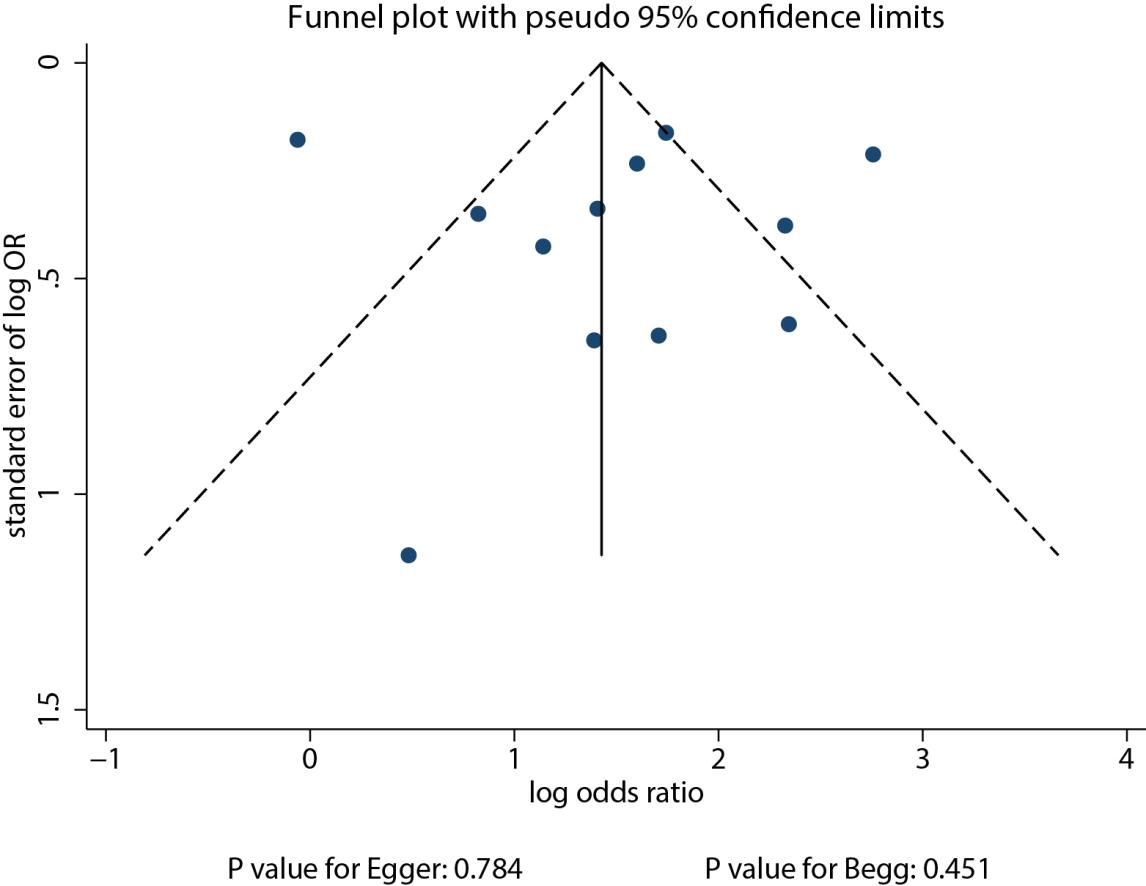


Figure S9. Funnel plot for large size of aneurysm on the risk of aneurysm rupture in UIA patients


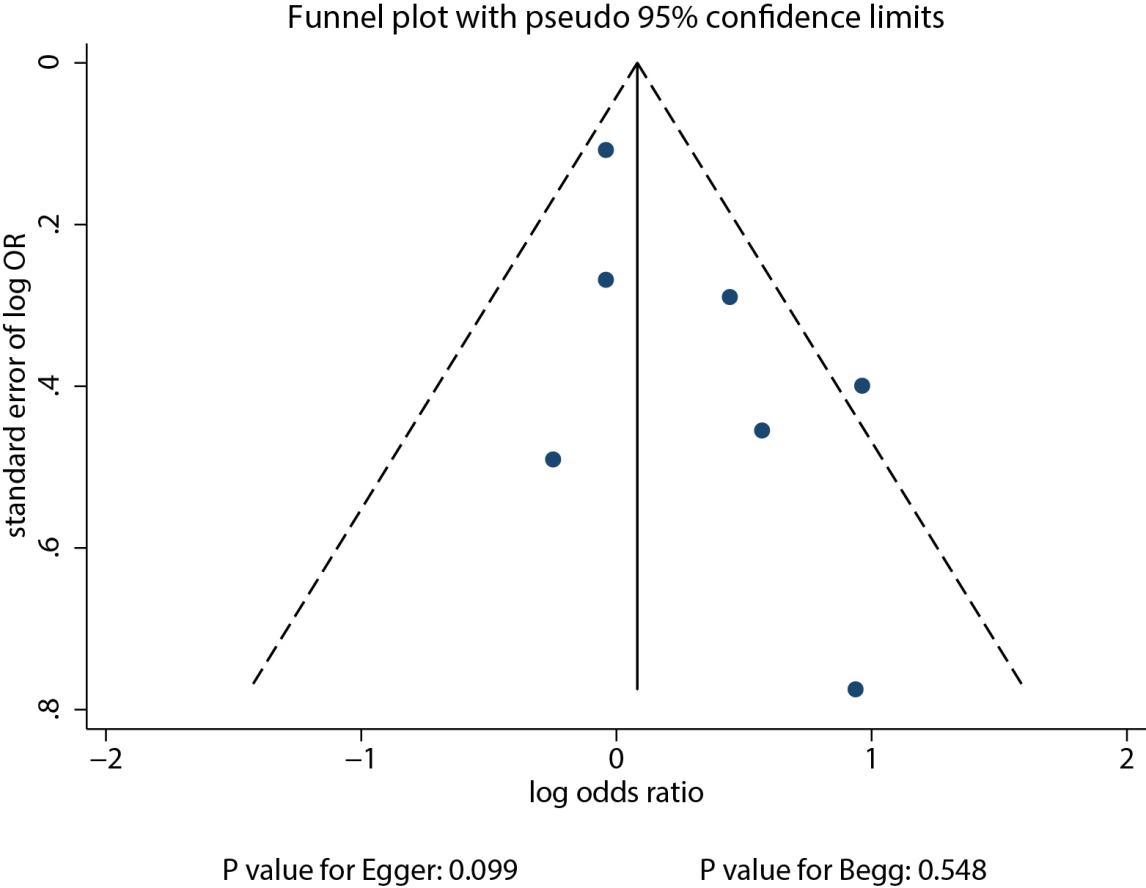


Figure S10. Funnel plot for multiple aneurysm on the risk of aneurysm rupture in UIA patients


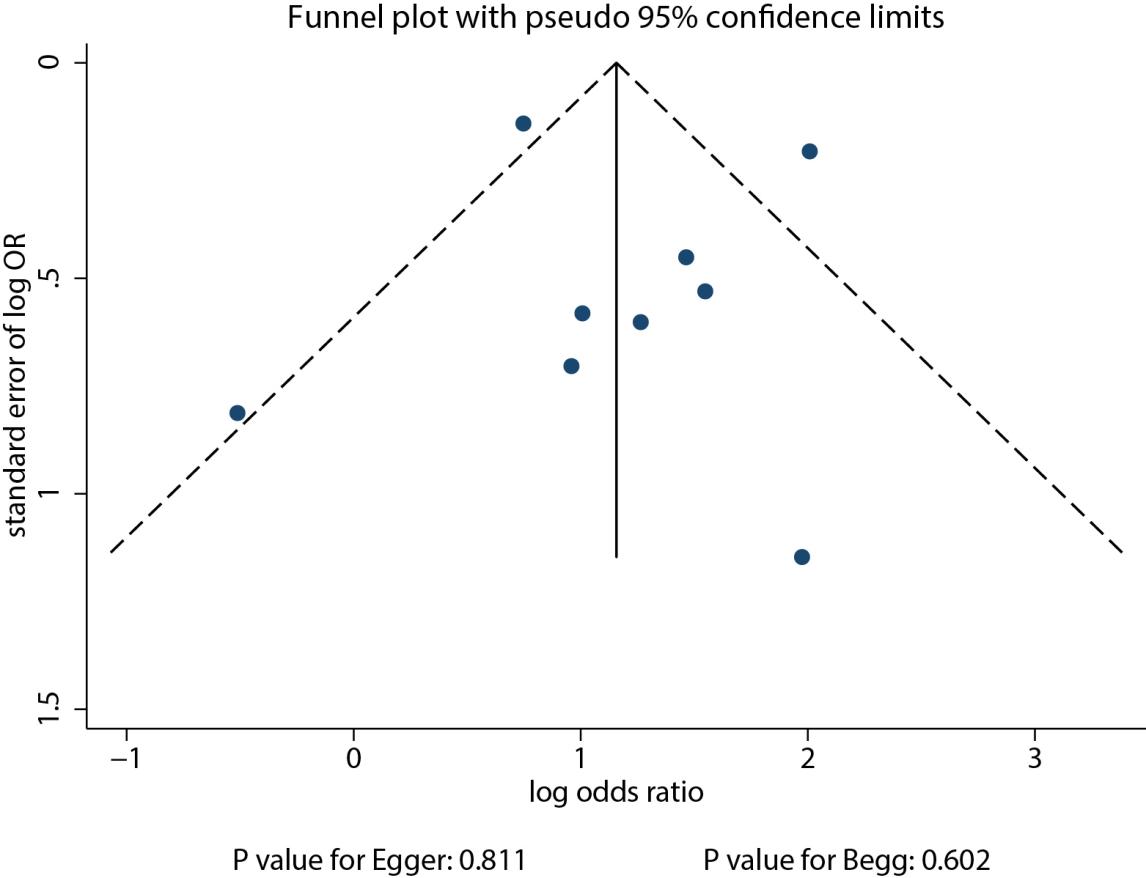


Figure S11. Funnel plot for ACA vs ICA on the risk of aneurysm rupture in UIA patients


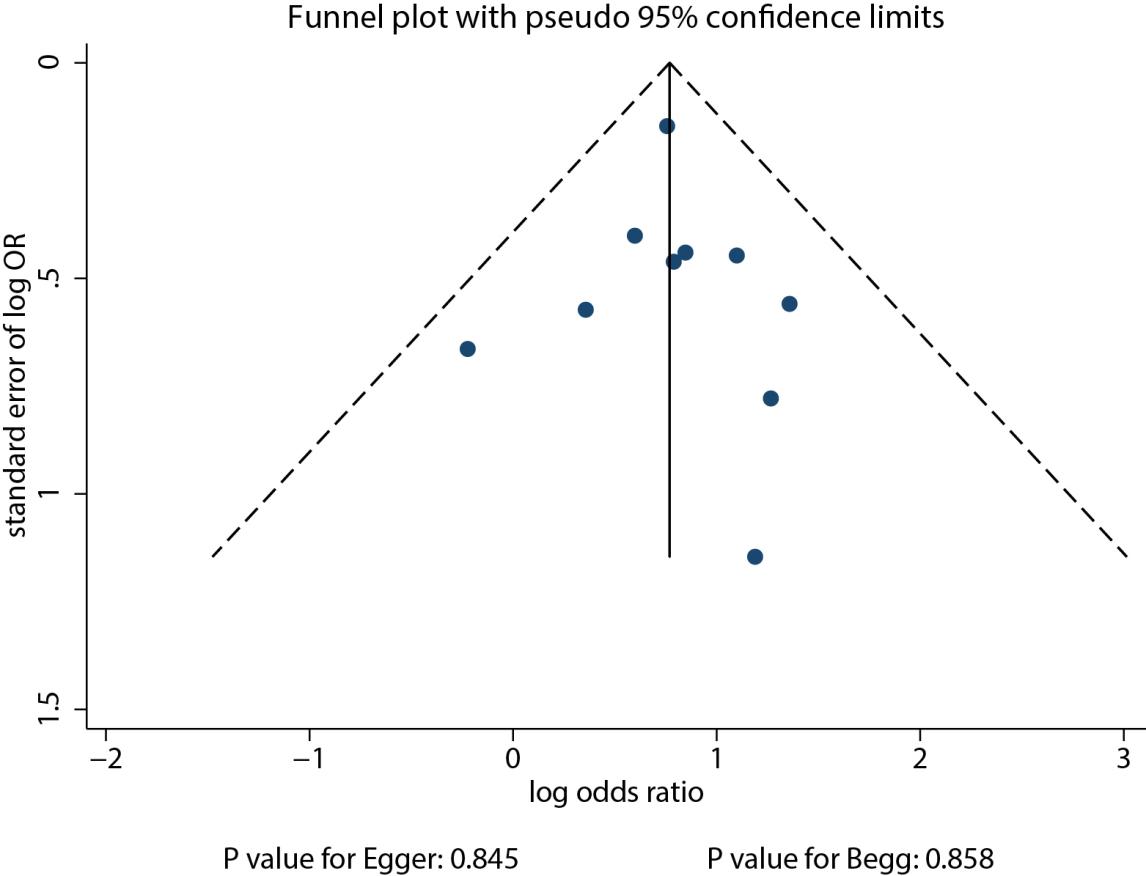


Figure S12. Funnel plot for MCA vs ICA on the risk of aneurysm rupture in UIA patients


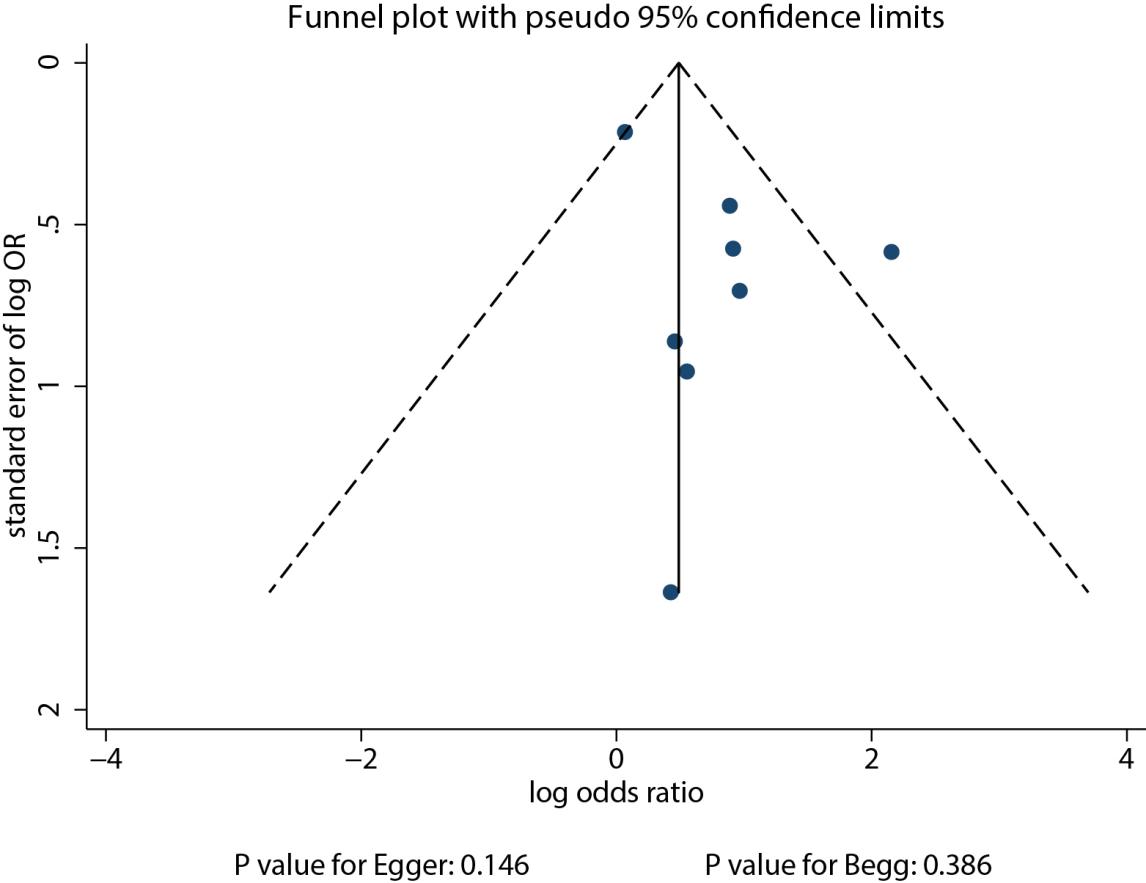


Figure S13. Funnel plot for VABA vs ICA on the risk of aneurysm rupture in UIA patients
